# Supplementary material for: Intraoperative Neuromonitoring Does Not Reduce the Risk of Temporary and Definitive Recurrent Laryngeal Nerve Damage during Thyroid Surgery: A Systematic Review and Meta-Analysis of Endoscopic Findings from 73,325 Nerves at Risk
Source: J Pers Med. 2023 Sep 23;13(10):1429. doi: 10.3390/jpm13101429 (PMC10607766; doi:10.3390/jpm13101429)
Supplement: Supplementary file 1 [file jpm-13-01429-s001.zip › supplementary material S1.pdf]

Database	Search date
Medline	January, the 19th, 2023
Embase	January, the 19th, 2023
Cochrane library	January, the 19th, 2023
Web Of Science	January, the 19th, 2023
Clinicaltrials.gov	January, the 19th, 2023
Scopus	January, the 19th, 2023

**Query**
("thyroidectomy"[MeSH Terms] OR "thyroidectomy"[All Fields] OR "thyroidectomies"[All Fields] OR ("hemithyroidectomies"[All Fields] OR "hemithyroidectomy"[All Fields]) OR ("lobectomies"[All Fields] OR "lobectomy"[All Fields]) OR ("isthmectomies"[All Fields] OR "isthmectomy"[All Fields]) OR ("loboisthmectomies"[All Fields] OR "loboisthmectomy"[All Fields])) AND ("laryngeal nerve"[All Fields] OR "recurrent nerve"[All Fields]) AND ("endoscopie"[All Fields] OR "endoscopy"[MeSH Terms] OR "endoscopy"[All Fields] OR "endoscopies"[All Fields] OR "endoscopy s"[All Fields] OR ("stroboscopies"[All Fields] OR "stroboscopy"[MeSH Terms] OR "stroboscopy"[All Fields]) OR ("fibroscopies"[All Fields] OR "fibroscopy"[All Fields]) OR "optic fiber"[All Fields] OR ("endoscope s"[All Fields] OR "endoscoped"[All Fields] OR "endoscopes"[MeSH Terms] OR "endoscopes"[All Fields] OR "endoscope"[All Fields] OR "endoscopical"[All Fields] OR "endoscopically"[All Fields] OR "endoscopy"[MeSH Terms] OR "endoscopy"[All Fields] OR "endoscopic"[All Fields]) OR ("laryngoscopy"[MeSH Terms] OR "laryngoscopy"[All Fields] OR "laryngoscopies"[All Fields]) OR ("laryngoscopy"[MeSH Terms] OR "laryngoscopy"[All Fields] OR "laryngoscopies"[All Fields]))
(thyroidectomy OR hemithyroidectomy OR lobectomy OR isthmectomy OR loboisthmectomy) AND ('laryngeal nerve' OR 'recurrent nerve') AND (endoscopy OR stroboscopy OR fibroscopy OR 'optic fiber' OR endoscopic OR laryngoscopy)
(thyroidectomy OR hemithyroidectomy OR lobectomy OR isthmectomy OR loboisthmectomy) AND ("laryngeal nerve" OR "recurrent nerve") AND (endoscopy OR stroboscopy OR fibroscopy OR "optic fiber" OR endoscopic OR laryngoscopy OR laryngoscopy) in Title Abstract Keyword - (Word variations have been searched)
(thyroidectomy OR hemithyroidectomy OR lobectomy OR isthmectomy OR loboisthmectomy) AND ("laryngeal nerve" OR "recurrent nerve") AND (endoscopy OR stroboscopy OR fibroscopy OR "optic fiber" OR endoscopic OR laryngoscopy OR laryngoscopy)
(thyroidectomy OR hemithyroidectomy OR lobectomy OR isthmectomy OR loboisthmectomy) AND ("laryngeal nerve" OR "recurrent nerve") AND (endoscopy OR stroboscopy OR fibroscopy OR "optic fiber" OR endoscopic OR laryngoscopy OR laryngoscopy)
ALL ((thyroidectomy OR hemithyroidectomy OR lobectomy OR isthmectomy OR loboisthmectomy) AND ("laryngeal nerve" OR "recurrent nerve") AND (endoscopy OR stroboscopy OR fibroscopy OR "optic fiber" OR endoscopic OR laryngoscopy OR laryngoscopy))
total hits
after duplicate removal

Items retrieved
729
1333
59
542
26
2883
5572
**3116**
