# Supplementary material for: Intraoperative Neuromonitoring Does Not Reduce the Risk of Temporary and Definitive Recurrent Laryngeal Nerve Damage during Thyroid Surgery: A Systematic Review and Meta-Analysis of Endoscopic Findings from 73,325 Nerves at Risk
Source: J Pers Med. 2023 Sep 23;13(10):1429. doi: 10.3390/jpm13101429 (PMC10607766; doi:10.3390/jpm13101429)
Supplement: Supplementary file 1 [file jpm-13-01429-s001.zip › Supplementary material S10.pdf]

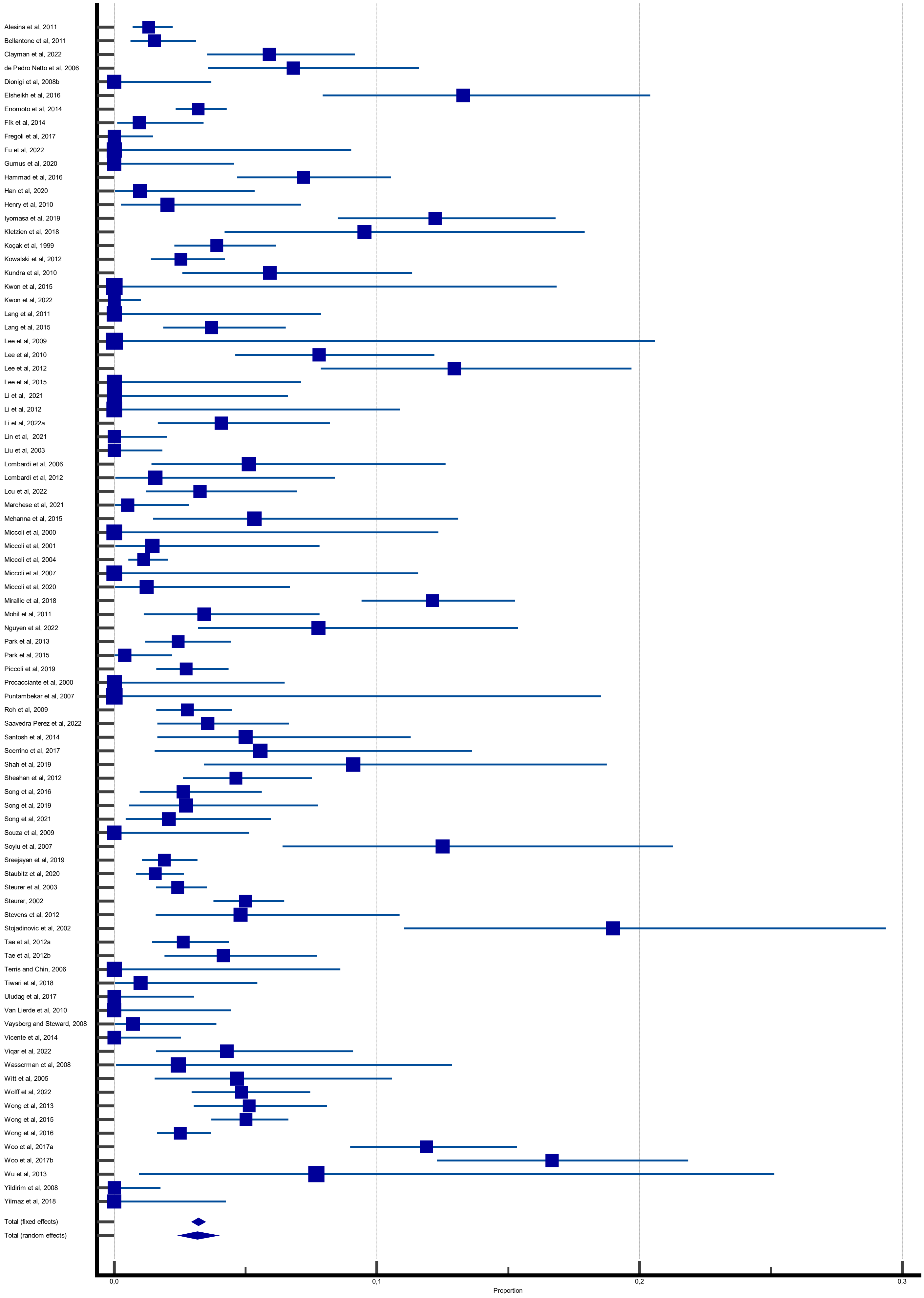

Alesina et al, 2011
Bellantone et al, 2011
Clayman et al, 2022
de Pedro Netto et al, 2006
Dionigi et al, 2008b
Elsheikh et al, 2016
Enomoto et al, 2014
Fík et al, 2014
Fregoli et al, 2017
Fu et al, 2022
Gumus et al, 2020
Hammad et al, 2016
Han et al, 2020
Henry et al, 2010
Iyomasa et al, 2019
Kletzien et al, 2018
Koçak et al, 1999
Kowalski et al, 2012
Kundra et al, 2010
Kwon et al, 2015
Kwon et al, 2022
Lang et al, 2011
Lang et al, 2015
Lee et al, 2009
Lee et al, 2010
Lee et al, 2012
Lee et al, 2015
Li et al, 2021
Li et al, 2012
Li et al, 2022a
Lin et al, 2021
Liu et al, 2003
Lombardi et al, 2006
Lombardi et al, 2012
Lou et al, 2022
Marchese et al, 2021
Mehanna et al, 2015
Miccoli et al, 2000
Miccoli et al, 2001
Miccoli et al, 2004
Miccoli et al, 2007
Miccoli et al, 2020
Mirallie et al, 2018
Mohil et al, 2011
Nguyen et al, 2022
Park et al, 2013
Park et al, 2015
Piccoli et al, 2019
Procacciante et al, 2000
Puntambekar et al, 2007
Roh et al, 2009
Saavedra-Perez et al, 2022
Santosh et al, 2014
Scerrino et al, 2017
Shah et al, 2019
Sheahan et al, 2012
Song et al, 2016
Song et al, 2019
Song et al, 2021
Souza et al, 2009
Soylu et al, 2007
Sreejayan et al, 2019
Staubitz et al, 2020
Steurer et al, 2003
Steurer, 2002
Stevens et al, 2012
Stojadinovic et al, 2002
Tae et al, 2012a
Tae et al, 2012b
Terris and Chin, 2006
Tiwari et al, 2018
Uludag et al, 2017
Van Lierde et al, 2010
Vaysberg and Steward, 2008
Vicente et al, 2014
Viqar et al, 2022
Wasserman et al, 2008
Witt et al, 2005
Wolff et al, 2022
Wong et al, 2013
Wong et al, 2015
Wong et al, 2016
Woo et al, 2017a
Woo et al, 2017b
Wu et al, 2013
Yildirim et al, 2008
Yilmaz et al, 2018
Total (fixed effects)
Total (random effects)
0,0
0,1
0,2
0,3
Proportion
