# Supplementary material for: Intraoperative Neuromonitoring Does Not Reduce the Risk of Temporary and Definitive Recurrent Laryngeal Nerve Damage during Thyroid Surgery: A Systematic Review and Meta-Analysis of Endoscopic Findings from 73,325 Nerves at Risk
Source: J Pers Med. 2023 Sep 23;13(10):1429. doi: 10.3390/jpm13101429 (PMC10607766; doi:10.3390/jpm13101429)
Supplement: Supplementary file 1 [file jpm-13-01429-s001.zip › Supplementary material S12.pdf]

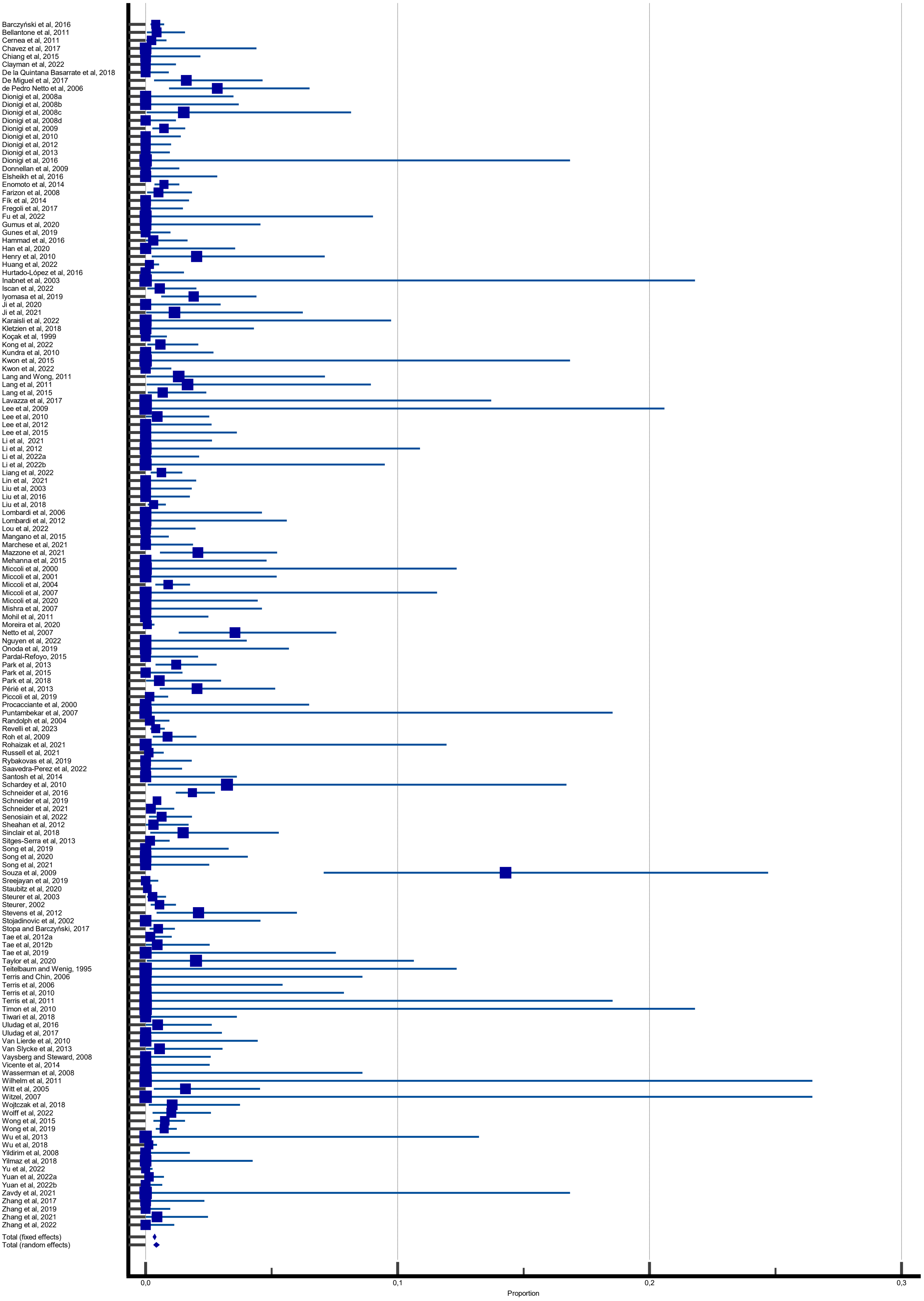

Barczyński et al, 2016
Bellantone et al, 2011
Cernea et al, 2011
Chavez et al, 2017
Chiang et al, 2015
Clayman et al, 2022
De la Quintana Basarrate et al, 2018
De Miguel et al, 2017
de Pedro Netto et al, 2006
Dionigi et al, 2008a
Dionigi et al, 2008b
Dionigi et al, 2008c
Dionigi et al, 2008d
Dionigi et al, 2009
Dionigi et al, 2010
Dionigi et al, 2012
Dionigi et al, 2013
Dionigi et al, 2016
Donnellan et al, 2009
Elsheikh et al, 2016
Enomoto et al, 2014
Farizon et al, 2008
Fík et al, 2014
Fregoli et al, 2017
Fu et al, 2022
Gumus et al, 2020
Gunes et al, 2019
Hammad et al, 2016
Han et al, 2020
Henry et al, 2010
Huang et al, 2022
Hurtado-López et al, 2016
Inabnet et al, 2003
Iscan et al, 2022
Iyomasa et al, 2019
Ji et al, 2020
Ji et al, 2021
Karaisli et al, 2022
Kletzien et al, 2018
Koçak et al, 1999
Kong et al, 2022
Kundra et al, 2010
Kwon et al, 2015
Kwon et al, 2022
Lang and Wong, 2011
Lang et al, 2011
Lang et al, 2015
Lavazza et al, 2017
Lee et al, 2009
Lee et al, 2010
Lee et al, 2012
Lee et al, 2015
Li et al, 2021
Li et al, 2012
Li et al, 2022a
Li et al, 2022b
Liang et al, 2022
Lin et al, 2021
Liu et al, 2003
Liu et al, 2016
Liu et al, 2018
Lombardi et al, 2006
Lombardi et al, 2012
Lou et al, 2022
Mangano et al, 2015
Marchese et al, 2021
Mazzone et al, 2021
Mehanna et al, 2015
Miccoli et al, 2000
Miccoli et al, 2001
Miccoli et al, 2004
Miccoli et al, 2007
Miccoli et al, 2020
Mishra et al, 2007
Mohil et al, 2011
Moreira et al, 2020
Netto et al, 2007
Nguyen et al, 2022
Onoda et al, 2019
Pardal-Refoyo, 2015
Park et al, 2013
Park et al, 2015
Park et al, 2018
Périé et al, 2013
Piccoli et al, 2019
Procacciante et al, 2000
Puntambekar et al, 2007
Randolph et al, 2004
Revelli et al, 2023
Roh et al, 2009
Rohaizak et al, 2021
Russell et al, 2021
Rybakovas et al, 2019
Saavedra-Perez et al, 2022
Santosh et al, 2014
Schardey et al, 2010
Schneider et al, 2016
Schneider et al, 2019
Schneider et al, 2021
Senosiain et al, 2022
Sheahan et al, 2012
Sinclair et al, 2018
Sitges-Serra et al, 2013
Song et al, 2019
Song et al, 2020
Song et al, 2021
Souza et al, 2009
Sreejayan et al, 2019
Staubitz et al, 2020
Steurer et al, 2003
Steurer, 2002
Stevens et al, 2012
Stojadinovic et al, 2002
Stopa and Barczyński, 2017
Tae et al, 2012a
Tae et al, 2012b
Tae et al, 2019
Taylor et al, 2020
Teitelbaum and Wenig, 1995
Terris and Chin, 2006
Terris et al, 2006
Terris et al, 2010
Terris et al, 2011
Timon et al, 2010
Tiwari et al, 2018
Uludag et al, 2016
Uludag et al, 2017
Van Lierde et al, 2010
Van Slycke et al, 2013
Vaysberg and Steward, 2008
Vicente et al, 2014
Wasserman et al, 2008
Wilhelm et al, 2011
Witt et al, 2005
Witzel, 2007
Wojtczak et al, 2018
Wolff et al, 2022
Wong et al, 2015
Wong et al, 2019
Wu et al, 2013
Wu et al, 2018
Yildirim et al, 2008
Yilmaz et al, 2018
Yu et al, 2022
Yuan et al, 2022a
Yuan et al, 2022b
Zavdy et al, 2021
Zhang et al, 2017
Zhang et al, 2019
Zhang et al, 2021
Zhang et al, 2022
Total (fixed effects)
Total (random effects)
0,0
0,1
0,2
0,3
Proportion
