# Supplementary material for: Intraoperative Neuromonitoring Does Not Reduce the Risk of Temporary and Definitive Recurrent Laryngeal Nerve Damage during Thyroid Surgery: A Systematic Review and Meta-Analysis of Endoscopic Findings from 73,325 Nerves at Risk
Source: J Pers Med. 2023 Sep 23;13(10):1429. doi: 10.3390/jpm13101429 (PMC10607766; doi:10.3390/jpm13101429)
Supplement: Supplementary file 1 [file jpm-13-01429-s001.zip › supplementary material S2.pdf]

Reference	Title	Study type
Alesina et al, 2011	Is minimally invasive, video-assisted thyroidectomy feasible in Graves' disease?	PCoS
Barczyński et al, 2016	The Overwhelming Majority but not All Motor Fibers of the Bifid Recurrent Laryngeal Nerve are Located in the Anterior Extralaryngeal Branch	PCoS
Bellantone et al, 2011	Is the identification of the external branch of the superior laryngeal nerve mandatory in thyroid operation? Results of a prospective randomized study	RCT
Cernea et al, 2011	Negative and positive predictive values of nerve monitoring in thyroidectomy	PCoS
Chavez et al, 2017	Comparative analysis between a bipolar vessel sealing and cutting device and the tie and suture technique in thyroidectomy: A randomized clinical trial	RCT
Chiang et al, 2015	Stimulating dissecting instruments during neuromonitoring of RLN in thyroid surgery	PCaS
Clayman et al, 2022	Human Amnion/Chorion Membrane May Reduce Transient Recurrent Laryngeal Nerve Injury During Thyroid Surgery	PCaS
a Quintana Basarrate et al,	Continuous monitoring of the recurrent laryngeal nerve	PCoS
De Miguel et al, 2017	Accuracy of transcutaneous laryngeal ultrasound for detecting vocal cord paralysis in the immediate postoperative period after total thyroidectomy	PCoS
de Pedro Netto et al, 2006	Voice and vocal self-assessment after thyroidectomy	PCoS
Dionigi et al, 2008a	Defining the learning curve for video-assisted thyroidectomy	PCoS
Dionigi et al, 2008b	The use of electrothermal bipolar vessel sealing system in minimally invasive video-assisted thyroidectomy (MIVAT)	PCaS
Dionigi et al, 2008c	Video-assisted thyroidectomy performed in a one-day surgery setting	PCaS
Dionigi et al, 2008d	What is the learning curve for intraoperative neuromonitoring in thyroid surgery?	PCaS
Dionigi et al, 2009	Postoperative laryngoscopy in thyroid surgery: proper timing to detect recurrent laryngeal nerve injury	PCoS
Dionigi et al, 2010	Surgical anatomy and neurophysiology of the vagus nerve (VN) for standardised intraoperative neuromonitoring (IONM) of the inferior laryngeal nerve (ILN) during	PCaS
Dionigi et al, 2012	The safety of energy-based devices in open thyroidectomy: A prospective, randomised study comparing the LigaSure™ (LF1212) and the Harmonic®	RCT

Dionigi et al, 2013	Parathyroid function after open thyroidectomy: A prospective randomized study for ligasure precise versus harmonic FOCUS	RCT
Dionigi et al, 2016	Transoral endoscopic thyroidectomy: preliminary experience in Italy	PCaS
Donnellan et al, 2009	Intraoperative laryngeal nerve monitoring during thyroidectomy	PCoS
Elsheikh et al, 2016	Voice Changes after Late Recurrent Laryngeal Nerve Identification Thyroidectomy	PCos
Enomoto et al, 2014	Recurrent laryngeal nerve palsy during surgery for benign thyroid diseases: risk factors and outcome analysis	PCos
Farizon et al, 2008	Intraoperative monitoring of the recurrent laryngeal nerve by vagal nerve stimulation in thyroid surgery	PCos
Fík et al, 2014	Minimally invasive video-assisted versus minimally invasive nonendoscopic thyroidectomy	PCoS
Fregoli et al, 2017	Postoperative Pain Evaluation after Robotic Transaxillary Thyroidectomy Versus Conventional Thyroidectomy: A Prospective Study	PCoS
Fu et al, 2022	The feasibility of laryngeal nerve protection during thyroidectomy using sternocleidomastoid intermuscular approach with intraoperative neuromonitoring: a case	PCaS
Gumus et al, 2020	Objective analysis of swallowing and functional voice outcomes after thyroidectomy: A prospective cohort study	PCoS
Gunes et al, 2019	Effect of intraoperative neuromonitoring on efficacy and safety using sugammadex in thyroid surgery: Randomized clinical trial	RCT
Hammad et al, 2016	A Prospective Study Comparing the Efficacy and Surgical Outcomes of Harmonic Focus Scalpel Versus LigaSure Small Jaw in Thyroid and Parathyroid Surgery	PCoS
Han et al, 2020	Functional Voice and Swallowing Outcome Analysis After Thyroid Lobectomy: Transoral Endoscopic Vestibular Versus Open Approach	PCoS
Henry et al, 2010	Functional voice outcomes after thyroidectomy: An assessment of the Dsyphonia Severity Index (DSI) after thyroidectomy	PCoS
Huang et al, 2022	Necessity of Routinely Testing the Proximal and Distal Ends of Exposed Recurrent Laryngeal Nerve During Monitored Thyroidectomy	PCoS
Hurtado-López et al, 2016	Efficacy of Intraoperative Neuro-Monitoring to Localize the External Branch of the Superior Laryngeal Nerve	PCoS
Inabnet et al, 2003	Neuromonitoring of the external branch of the superior laryngeal nerve during minimally invasive thyroid surgery under local anesthesia: a prospective study of 10 patients	PCoS
Iscan et al, 2022	Is craniocaudal dissection of recurrent laryngeal nerve safer than lateral approach: a prospective randomized study comparing both techniques by using continuous	PCoS
Iyomasa et al, 2019	Laryngeal and vocal alterations after thyroidectomy	PCoS

Ji et al, 2020	Feasibility and efficacy of intraoperative neural monitoring in remote access robotic and endoscopic thyroidectomy	PCoS
Ji et al, 2021	Neural Monitoring of the External Branch of the Superior Laryngeal Nerve During Transoral Thyroidectomy	PCaS
Karaisli et al, 2022	Comparison of stimulating dissector and intermittent stimulating probe for the identification of recurrent laryngeal nerve in reoperative setting	PCaS
Kletzien et al, 2018	Comparison Between Patient-Perceived Voice Changes and Quantitative Voice Measures in the First Postoperative Year After Thyroidectomy: A Secondary	PCoS
Koçak et al, 1999	Evaluation of vocal cord function after thyroid surgery	PCoS
Kong et al, 2022	Comparison of a Handheld Device vs Endotracheal Tube-Based Neuromonitoring for Recurrent Laryngeal Nerve Stimulation	PCoS
Kowalski et al, 2012	Total thyroidectomy with ultrasonic scalpel: A multicenter, randomized controlled trial	RCT
Kundra et al, 2010	Laryngoscopic techniques to assess vocal cord mobility following thyroid surgery	PCaS
Kwon et al, 2015	Role of charcoal tattooing in localization of recurred papillary thyroid carcinoma: Initial experiences	PCaS
Kwon et al, 2022	Clinical Significance of the Preoperative Thyroidectomy-Related Voice Questionnaire Score in Thyroid Surgery	PCaS
Lang and Wong, 2011	Feasibility on the use of intraoperative vagal nerve stimulation in gasless, transaxillary endoscopic, and robotic-assisted thyroidectomy	PCaS
Lang et al, 2011	A comparison of surgical outcomes between endoscopic and robotically assisted thyroidectomy: the authors' initial experience	PCaS
Lang et al, 2015	Pain and surgical outcomes with and without neck extension in standard open thyroidectomy: A prospective randomized trial	RCT
Lavazza et al, 2017	Transoral endoscopic thyroidectomy: Preliminary experience in Italy	PCaS
Lee et al, 2009	Postauricular and axillary approach endoscopic neck surgery: a new technique	PCaS
Lee et al, 2010	Outcomes of 109 patients with papillary thyroid carcinoma who underwent robotic total thyroidectomy with central node dissection via the bilateral axillo-breast approach	PCaS
Lee et al, 2012	Postoperative functional voice changes after conventional open or robotic thyroidectomy: a prospective trial	PCoS
Lee et al, 2015	The Efficacy of Intraoperative Neuromonitoring During Robotic Thyroidectomy: A Prospective, Randomized Case-Control Evaluation	RCT
Li et al, 2021	Advantages of intraoperative nerve monitoring in endoscopic thyroidectomy for papillary thyroid carcinoma	PCaS

Li et al, 2012	A voice acoustic analysis of thyroid adenoma patients after a unilateral thyroid lobectomy	PCoS
Li et al, 2022a	Comparison of quality of life and cosmetic result between open and transaxillary endoscopic thyroid lobectomy for papillary thyroid microcarcinoma survivors: A single-	PCoS
Li et al, 2022b	Thyroidectomy using a single-port cervico-mental angle approach	PCaS
Liang et al, 2022	Thyroidectomy for thyroid cancer via transareola single-site endoscopic approach: results of a case-match study with large-scale population	PCoS
Lin et al, 2021	Comparative study of gasless endoscopic selective lateral neck dissection via the anterior chest approach versus conventional open surgery for papillary thyroid carcinoma	PCoS
Liu et al, 2003	Minimally invasive low-collar incision in thyroid lobectomy	PCaS
Liu et al, 2016	Exclusive real-time monitoring during recurrent laryngeal nerve dissection in conventional monitored thyroidectomy	PCaS
Liu et al, 2018	Laryngeal nerve morbidity in 1.273 central node dissections for thyroid cancer	PCaS
Lombardi et al, 2006	Voice and swallowing changes after thyroidectomy in patients without inferior laryngeal nerve injuries	PCoS
Lombardi et al, 2012	Prospective electromyographic evaluation of functional postthyroidectomy voice and swallowing symptoms.	PCaS
Lou et al, 2022	Transoral Endoscopic Thyroidectomy Vestibular Approach (TOETVA) in the Perioperative Mode of Day Ward	PCaS
Mangano et al, 2015	Continuous intraoperative neuromonitoring in thyroid surgery: Safety analysis of 400 consecutive electrode probe placements with standardized procedures	PCaS
Marchese et al, 2021	Neck complaints before and after uncomplicated thyroidectomy: prevalence, postoperative outcome and relationships with thyroid weight and reflux like symptoms	PCaS
Mazzone et al, 2021	Continuous Intraoperative Nerve Monitoring in Thyroid Surgery: Can Amplitude Be a Standardized Parameter?	PCaS
Mehanna et al, 2015	Effect of endotracheal tube size on vocal outcomes after thyroidectomy: A randomized clinical trial	RCT
Miccoli et al, 2000	Minimally invasive video-assisted surgery of the thyroid: a preliminary report.	PCaS
Miccoli et al, 2001	Impact of harmonic scalpel on operative time during video-assisted thyroidectomy	PCoS
Miccoli et al, 2004	Minimally invasive video-assisted thyroidectomy: five years of experience	PCaS
Miccoli et al, 2007	Video-assisted central compartment lymphadenectomy in a patient with a positive RET oncogene: initial experience	PCaS

Miccoli et al, 2020	Minimally invasive video-assisted thyroidectomy	PCaS
Mirallie et al, 2018	Does intraoperative neuromonitoring of recurrent nerves have an impact on the postoperative palsy rate? Results of a prospective multicenter study	RCT
Mishra et al, 2007	The external laryngeal nerve in thyroid surgery: The 'no more neglected' nerve	PCoS
Mohil et al, 2011	Recurrent laryngeal nerve and voice preservation: routine identification and appropriate assessment - two important steps in thyroid surgery	PCaS
Moreira et al, 2020	Investigation of recurrent laryngeal palsy rates for potential associations during thyroidectomy	PCaS
Moreno Llorente et al, 2023	Transoral endoscopic thyroidectomy vestibular approach: Results after 53 first cases [Tiroidectomía endoscópica transoral por vía vestibular: resultados de los 53 primeros	PCaS
Netto et al, 2007	Vocal fold immobility after thyroidectomy with intraoperative recurrent laryngeal nerve monitoring	PCoS
Nguyen et al, 2022	Comparison of Transoral Endoscopic Thyroidectomy Vestibular Approach and Conventional Open Thyroidectomy in Benign Thyroid Tumors	PCaS
Onoda et al, 2019	Continuous intraoperative neuromonitoring for thyroid cancer surgery: A prospective study	PCoS
Pardal-Refoyo, 2015	Usefulness of neuromonitoring in thyroid surgery	PCaS
Park et al, 2013	How can we screen voice problems effectively in patients undergoing thyroid surgery?	PCaS
Park et al, 2015	Robotic thyroidectomy learning curve for beginning surgeons with little or no experience of endoscopic surgery	PCoS
Park et al, 2018	Long-term voice outcome after thyroidectomy using energy based devices	PCoS
Périé et al, 2013	Value of recurrent laryngeal nerve monitoring in the operative strategy during total thyroidectomy and parathyroidectomy	PCaS
Piccoli et al, 2019	Evolution Strategies in Transaxillary Robotic Thyroidectomy: Considerations on the First 449 Cases Performed	PCaS
Procacciante et al, 2000	Palpatory method used to identify the recurrent laryngeal nerve during thyroidectomy	PCaS
Puntambekar et al, 2007	Endoscopic thyroidectomy: Our technique	PCaS
Randolph et al, 2004	Recurrent laryngeal nerve identification and assessment during thyroid surgery: laryngeal palpation	PCaS
Revelli et al, 2023	Is There Any Reliable Predictor of Functional Recovery Following Post-thyroidectomy Vocal Fold Paralysis?	PCoS

Roh et al, 2009	Recurrent laryngeal nerve paralysis in patients with papillary thyroid carcinomas: evaluation and management of resulting vocal dysfunction	PCaS
Rohaizak et al, 2021	Vagus Nerve Injury during Continuous Intraoperative Neuromonitoring (cIONM) for Thyroid Surgery: Assessment of Severity	PCoS
Russell et al, 2021	Transoral Thyroidectomy: Safety and Outcomes of 200 Consecutive North American Cases	PCoS
Rybakovas et al, 2019	Recurrent laryngeal nerve injury assessment by intraoperative laryngeal ultrasonography: A prospective diagnostic test accuracy study	PCoS
Saavedra-Perez et al, 2022	Thyroidectomy via unilateral axillo-breast approach (UABA) with gas insufflation: prospective multicentre European study	PCoS
Santosh et al, 2014	Capsular dissection method in thyroidectomy	PCaS
Scerrino et al, 2017	Long-term esophageal motility changes after thyroidectomy: Associations with aerodigestive disorders	PcoS
Schardey et al, 2010	Invisible scar endoscopic dorsal approach thyroidectomy: a clinical feasibility study.	PCoS
Schneider et al, 2016	Dynamics of loss and recovery of the nerve monitoring signal during thyroidectomy predict early postoperative vocal fold function	PCoS
Schneider et al, 2019	Complete and incomplete recurrent laryngeal nerve injury after thyroid and parathyroid surgery: Characterizing paralysis and paresis	PCoS
Schneider et al, 2021	Recurrent laryngeal nerve preservation strategies in pediatric thyroid oncology: Continuous vs. intermittent nerve monitoring	PCoS
Senosiain et al, 2022	Utility of the continuous intraoperative neuromonitoring in the prevention of the recurrent laryngeal nerve paralysis during thyroid surgery. A prospective observational study	PCoS
Shah et al, 2019	Comparison of transcutaneous laryngeal ultrasound with video laryngoscope for assessing the vocal cord mobility in patients undergoing thyroid surgery	PCaS
Sheahan et al, 2012	Risk factors for recurrent laryngeal nerve neuropraxia postthyroidectomy	PCoS
Sinclair et al, 2018	Noninvasive, tube-based, continuous vagal nerve monitoring using the laryngeal adductor reflex: Feasibility study of 134 nerves at risk	PCoS
Sitges-Serra et al, 2013	Prospective study on loss of signal on the first side during neuromonitoring of the recurrent laryngeal nerve in total thyroidectomy	PCoS
Song et al, 2016	Long-Term Voice Outcomes after Robotic Thyroidectomy	PCoS
Song et al, 2019	Comparison of postoperative voice outcomes after postauricular facelift robotic hemithyroidectomy and conventional transcervical hemithyroidectomy	PCoS
Song et al, 2020	Voice outcomes of transoral robotic thyroidectomy: Comparison with conventional trans-cervical thyroidectomy	PCoS

Song et al, 2021	Health-related quality of life after transoral robotic thyroidectomy in papillary thyroid carcinoma	PCoS
Souza et al, 2009	Laryngeal vocal and endoscopic alterations after thyroidectomy under local anesthesia and hypnosedation	PCaS
Soylu et al, 2007	The evaluation of the causes of subjective voice disturbances after thyroid surgery	PCoS
Sreejayan et al, 2019	Inferior Approach: a Safe Method for Identification of Recurrent Laryngeal Nerve During Thyroidectomy	PCaS
Staubitz et al, 2020	Effect of intraoperative nerve monitoring on postoperative vocal cord palsy rates after thyroidectomy: European multicentre registry-based study	PCaS
Steurer et al, 2003	Functional Laryngeal Results after Thyroidectomy and Extensive Recurrent Laryngeal Nerve Dissection Without Neuromonitoring - An Analysis of More Than 1 000	PCoS
Steurer, 2002	Advantages of recurrent laryngeal nerve identification in thyroidectomy and parathyroidectomy and the importance of preoperative and postoperative laryngoscopic	PCoS
Stevens et al, 2012	The impact of recurrent laryngeal neuromonitoring on multi-dimensional voice outcomes following thyroid surgery	PCoS
Stojadinovic et al, 2002	Prospective functional voice assessment in patients undergoing thyroid surgery	PCoS
Stopa and Barczyński, 2017	Prognostic value of intraoperative neural monitoring of the recurrent laryngeal nerve in thyroid surgery	PCoS
Tae et al, 2012a	Early surgical outcomes of robotic thyroidectomy by a gasless unilateral axillo-breast or axillary approach for papillary thyroid carcinoma: 2 years' experience	PCoS
Tae et al, 2012b	Functional voice and swallowing outcomes after robotic thyroidectomy by a gasless unilateral axillo-breast approach: comparison with open thyroidectomy	PCoS
Tae et al, 2019	Early experience of transoral thyroidectomy: Comparison of robotic and endoscopic procedures	PCaS
Taylor et al, 2020	Changes in Tracheal Tube Cuff Pressure and Recurrent Laryngeal Nerve Conductivity During Thyroid Surgery	PCaS
Teitelbaum and Wenig, 199[illegible]	Superior laryngeal nerve injury from thyroid surgery	PCoS
Terris and Chin, 2006	Clinical implementation of endoscopic thyroidectomy in selected patients	PCaS
Terris et al, 2006	Ultrasonic technology facilitates minimal access thyroid surgery	PCaS
Terris et al, 2010	Reoperative thyroidectomy for benign thyroid disease	PCoS
Terris et al, 2011	Robotic facelift thyroidectomy: II. Clinical feasibility and safety	PCaS

Timon et al, 2010	Investigation of the impact of thyroid surgery on vocal tract steadiness	PCaS
Tiwari et al, 2018	Prospective study of complications following surgery for thyroid malignancy: A tertiary cancer care centre experience	PCoS
Uludag et al, 2016	Contribution of intraoperative neural monitoring to preservation of the external branch of the superior laryngeal nerve: a randomized prospective clinical trial	RCT
Uludag et al, 2017	Effect of energy-based devices on voice quality after total thyroidectomy	RCT
Van Lierde et al, 2010	Impact of thyroidectomy without laryngeal nerve injury on vocal quality characteristics: an objective multiparameter approach	PCoS
Van Slycke et al, 2013	Initial experience with S-shaped electrode for continuous vagal nerve stimulation in thyroid surgery	PCoS
Vaysberg and Steward, 200[illegible]	Minimally invasive video-assisted thyroidectomy	PCaS
Vicente et al, 2014	Voice outcomes after total thyroidectomy, partial thyroidectomy, or non-neck surgery using a prospective multifactorial assessment	PCoS
Viqar et al, 2022	Early and late complications of thyroidectomy: A descriptive cohort study in Rawalpindi	PCaS
Wasserman et al, 2008	Determination of the function of the internal branch of the superior laryngeal nerve after thyroidectomy	PCaS
Wilhelm et al, 2011	Endoscopic minimally invasive thyroidectomy (eMIT): a prospective proof-of-concept study in humans.	PCoS
Witt et al, 2005	Recurrent laryngeal nerve electrophysiologic monitoring in thyroid surgery: The standard of care?	PCaS
Witzel, 2007	The axillary access in unilateral thyroid resection	PCaS
Wojtczak et al, 2018	Evaluating the introduction of intraoperative neuromonitoring of the recurrent laryngeal nerve in thyroid and parathyroid surgery	PCaS
Wolff et al, 2022	Factors Associated With Injury to Recurrent Laryngeal Nerve in Patients Undergoing Surgery for Thyroid Cancer: A Single-centre Study Using Translaryngeal Ultrasound	PCoS
Wong et al, 2013	A prospective, assessor-blind evaluation of surgeon-performed transcutaneous laryngeal ultrasonography in vocal cord examination before and after thyroidectomy	PCaS
Wong et al, 2015	Assessing the Validity of Transcutaneous Laryngeal Ultrasonography (TLUSG) After Thyroidectomy: What Factors Matter?	PCaS
Wong et al, 2016	Determining the Learning Curve of Transcutaneous Laryngeal Ultrasound in Vocal Cord Assessment by CUSUM Analysis of Eight Surgical Residents: When to	PCaS
Wong et al, 2019	Vocal Cord Palsies Missed by Transcutaneous Laryngeal Ultrasound (TLUSG): Do They Experience Worse Outcomes?	PCaS

Woo et al, 2017a	A Novel Gel Pad Laryngeal Ultrasound for Vocal Cord Evaluation	PCaS
Woo et al, 2017b	Comparison of ultrasound frequency in laryngeal ultrasound for vocal cord evaluation	PCaS
Wu et al, 2013	Video-assisted selective lateral neck dissection for papillary thyroid carcinoma	PCoS
Wu et al, 2018	Staged Thyroidectomy: A Single Institution Perspective	PCaS
Yildirim et al, 2008	Ultrasonic harmonic scalpel in total thyroidectomies	PCaS
Yilmaz et al, 2018	An analysis on aerodynamic and acoustic changes after thyroidectomy	PCoS
Yu et al, 2022	Effects of Intraoperative Neural Tunnel in Protecting Recurrent Laryngeal Nerve: Experiences in Open, Trans Breast, and Transoral Endoscopic Thyroidectomy	PCoS
Yuan et al, 2022a	Total thyroidectomy versus hemithyroidectomy with intraoperative radiofrequency ablation for unilateral thyroid cancer with contralateral nodules: A propensity score	PCoS
Yuan et al, 2022b	Visual identification and neuromonitoring vs. no sighting the external branch of the superior laryngeal nerve in thyroid surgery: a randomized clinical trial	PCoS
Zavdy et al, 2021	Intraoperative Ultrasonographic Assessment of Vocal Cord motion under sedation, following paediatric thyroidectomy in the Era of COVID-19: A double-blinded	PCaS
Zhang et al, 2017	Percutaneous probe stimulation for intraoperative neuromonitoring in total endoscopic thyroidectomy: A preliminary experience	PCoS
Zhang et al, 2019	Central Lymph Node Dissection by Endoscopic Bilateral Areola Versus Open Thyroidectomy	PCaS
Zhang et al, 2021	Drawbacks of neural monitoring troubleshooting algorithms in transoral endoscopic thyroidectomy	PCaS
Zhang et al, 2022	Clinical Experience of Use of Percutaneous Continuous Nervemonitoring in Robotic Bilateral Axillo-Breast Thyroid Surgery	PCoS

Evidence level according to OCEBM	NIH-SQAT Rating (for non-RCT) or overall risk of bias (for RCT)	Procedures included (n)	Procedure type (n)				Access type (with *n* if multiple types in the study)	IONM use (with *n* if used only on several patients in a single study)
			TT	ST	CT	L		
3	fair	497	482	9	6	0	endoscopic (n=157), open	no
3	good	1268	1232	0	18	18	open	yes
2	intermediate	289	170	0	0	119	open	no
3	good	447	421	26	0	0	open	yes
2	intermediate	41	39	2	0	0	open	yes
4	fair	100	70	0	0	30	n/a	yes
4	fair	201	101	0	0	100	Open	No
3	good	248	229	0	19	0	n/a	yes
3	good	93	93	0	0	0	open	yes
3	good	88	88	0	0	0	open	no
3	fair	67	37	0	0	30	endoscopic	yes
4	fair	63	35	0	0	28	endoscopic	no
4	fair	43	23	0	0	20	endoscopic	yes
4	fair	152	152	0	0	0	open	yes
3	good	434	391	0	0	43	n/a	yes
4	fair	140	123	0	0	17	open	yes
2	intermediate	182	182	0	0	0	open	yes

2	intermediate	199	183	0	0	16	open	yes
4	fair	15	5	0	0	10	endoscopic	yes
3	fair	210	63	0	0	147	n/a	yes
3	fair	64	51	0	0	13	open	no
3	fair	844	310	514	0	0	open	no
3	fair	195	195	0	0	0	open	yes
3	fair	131	81	0	0	50	endoscopic	no
3	fair	124	n/a	n/a	n/a	n/a	robot	no
4	fair	39	0	0	0	39	open	yes
3	fair	40	39	0	0	1	open	no
2	intermediate	203	170	0	0	33	open	yes
3	fair	294	44	0	43	149	open	no
3	fair	102	0	0	0	102	50 open 52 endoscopic	no
3	fair	62	37	0	0	25	Open	no
3	fair	796	564	0	0	232	open	yes
3	good	148	92	0	0	56	open	yes
3	good	10	5	0	0	5	endoscopic	yes
3	fair	198	158	0	0	40	open	yes
3	good	151	111	0	0	40	open	no

3	fair	104	27	0	0	77	Robotic (n=82) endoscopic	yes
4	good	76	11	0	0	65	Robotic (n=61) endoscopic	yes
4	fair	32	0	0	32	0	open	yes
3	good	42	42	0	0	0	Open	no
3	fair	218	69	149	0	0	open	no
3	good	249	84	0	22	153	open	yes
2	intermediate	261	261	0	0	0	open	no
4	fair	104	31	8	0	65	open	no
4	good	10	10	0	0	0	open	no
4	good	180	180	0	0	0	open	no
4	fair	60	16	1	0	43	endoscopic (n=52) Robotic	yes
4	fair	46	14	0	0	32	endoscopic (n=39) robotic (n=7)	15 with nim
2	low	180	117	0	1	62	open	no
4	good	15	10	0	0	5	endoscopic	yes
4	good	10	6	1	0	2	endoscopic	no
4	good	109	109	0	0	0	Robotic	no
3	fair	88	51	0	0	37	open (n=46), robotic (n=42)	no
2	intermediate	50	50	0	0	0	robotic	yes (n=25), no (n=25)
4	fair	69	69	0	0	0	endoscopic	42 con nim

3	fair	32	0	0	0	32	open	no
3	fair	172	0	0	0	172	open (n=99) endoscopic (n=73)	no
4	fair	30	7	0	0	23	endoscopic	yes
3	fair	400	400	0	0	0	open (n=200) endoscopic	yes
3	fair	91	91	0	0	0	endoscopic (n=31) open (n=60)	no
4	fair	200	0	0	0	200	open	no
4	good	120	102	0	0	18	open	yes
4	fair	1119	n/a	n/a	n/a	n/a	open	yes
3	fair	39	39	0	0	0	open or endoscopic (n unknown)	no
4	fair	32	32	0	0	0	open or endoscopic (n unknown)	no
4	fair	157	27	0	0	130	endoscopic	no
4	good	211	189	0	0	22	open	yes
4	fair	97	97	0	0	0	open	no
4	good	108	87	0	0	21	open	yes
2	low	49	26	0	0	23	open	no
4	fair	27	1	0	2	24	endoscopic	no
3	fair	116	17	0	0	35	endoscopic	no
4	good	579	312	0	0	267	endoscopic	no
4	fair	15	15	0	0	0	endoscopic	no

4	fair	67	15	0	0	51	endoscopic	no
2	intermediate	1328	1315	0	0	5	open	yes (n=807) no (512)
4	fair	46	32	0	0	14	open	no
4	fair	100	41	5	6	48	open	no
4	good	1003	565	0	0	438	open	yes
4	good	53	11	0	0	42	endoscopic	yes
3	fair	104	65	0	0	39	open	yes
4	fair	78	12	0	0	66	open (n=31) endoscopic (n=47)	no
3	fair	43	20	0	0	23	open	yes
4	fair	91	84	0	0	7	open	yes
4	fair	242	169	0	0	73	open	no
3	fair	125	125	0	0	0	robotic	no
3	fair	92	92	0	0	0	open	yes
4	good	100	91	0	0	0	open	yes
4	fair	449	172	0	0	277	robotic	no
4	fair	45	10	0	0	35	open	no
4	fair	15	3	4	0	8	endoscopic	no
4	fair	449	n/a	n/a	n/a	n/a	open	yes
3	fair	51	46	0	0	5	open	yes

4	good	319	256	0	21	42	open	no
3	fair	18	11	0	0	7	open	yes
3	good	533	240	0	0	293	open (n=333) endoscopic	yes
3	good	112	54	0	2	56	open	yes
3	fair	253	0	0	0	253	endoscopic	no
4	fair	80	13	7	0	60	open	no
3	fair	36	36	0	0	0	endoscopic (n=9), open (n=27)	no
3	fair	30	0	1	0	29	endoscopic	yes
3	fair	785	N/A	n/a	n/a	n/a	open	yes
3	good	4707	3302	0	0	1405	open	yes
3	fair	258	183	75	0	0	open	yes
3	fair	248	171	0	15	62	Open	yes
4	fair	45	21	0	0	24	open	no
3	good	209	113	0	7	89	open	no
3	good	100	27	0	0	64	open	yes
3	fair	290	290	0	0	0	open	Yes
3	Fair	124	105	0	0	19	open (n=70) robotic (n=54)	no
3	good	110	0	0	0	110	open (n=68), robotic (n=42)	no
3	fair	89	0	0	0	89	open (n=47) robotic (n=42)	yes

3	good	114	30	0	0	84	open (n=57) robotic (n=57)	no
4	Fair	35	n/a	0	0	n/a	open	no
3	fair	48	40	0	0	8	open	no
4	good	393	343	0	0	50	open	no
4	good	4598	4598	0	0	0	open	yes (4182), no (416)
3	fair	624	362	0	56	206	open	no
3	good	608	308	83	51	122	open	no
3	fair	91	52	0	0	39	open	yes (n=39), no (-n=52)
3	fair	50	29	0	0	21	open	no
3	good	500	500	0	0	0	open	yes
3	good	301	233	0	0	68	robotic (n=75) open (n=226)	no
3	good	111	88	0	0	23	open (n=61) robotic (n=50)	no
4	good	37	10	0	0	27	endoscopic(14) robotic (23)	yes
4	good	32	18	0	2	12	open	yes
3	fair	20	6	2	0	12	open	no
4	fair	36	5	0	1	30	endoscopic	no
4	fair	44	22	0	4	18	open	yes
3	fair	45	0	0	45	0	open	yes
4	fair	18	1	0	4	13	robotic	yes

4	good	10	5	0	0	5	open	yes
3	fair	50	50	0	0	0	open	no
2	intermediate	133	77	0	0	56	open	yes
2	intermediate	60	60	0	0	0	open	no
3	good	44	37	0	0	7	open	no
3	good	100	80	0	0	20	open	yes
4	fair	86	50	0	5	31	endoscopic	no
3	good	112	54	0	0	35	open	no
4	fair	70	70	0	0	0	open	no
4	fair	33	4	0	3	26	open	no
3	good	8	4	0	0	4	endoscopic	yes
4	fair	136	54	0	0	82	open	no (n=107)<br>yes (n=83)
4	fair	12	0	0	0	12	endoscopic	yes
4	good	101	55	15	0	14	open	yes
3	fair	196	196	0	0	0	open	no
4	fair	204	115	0	15	74	open	no
4	good	581	336	0	40	205	open	no
4	fair	640	343	0	38	223	open	no
4	good	1196	667	0	54	390	open	yes

4	fair	278	130	4	0	124	open (n=239), robotic(n=26	no
4	fair	162	79	3	0	72	open (n=140), robotic(n=11	no
3	fair	26	0	0	0	26	endoscopic	no
4	good	803	803	0	0	0	open	yes
4	fair	104	104	0	0	0	open	no
3	fair	44	34	7	0	3	open	no
3	fair	929	388	0	0	541	open (n=315) endoscopic	yes
3	fair	382	191	0	0	191	open	yes
3	good	278	278	0	0	0	open	yes
4	good	15	5	0	2	8	open	yes
3	good	132	34	0	0	98	endoscopic	yes
4	fair	400	N/A	n/a	n/a	n/a	open (n=200) endoscopic	yes
4	good	179	44	0	0	135	endoscopic	yes
3	fair	304	304	0	0	0	Robotic	yes

procedures with IONM			procedures without IONM			
**damaged RLN (n)**	**healthy RLN (n)**	**total RLN (n)**	**damaged RLN (n)**	**healthy RLN (n)**	**total RLN (n)**	**RLN at risk (n)**
0	0	0	13	981	994	994
62	2438	2500	0	0	0	2500
0	0	0	7	452	459	459
10	858	868	0	0	0	868
4	78	82	0	0	0	82
1	167	168	0	0	0	168
0	0	0	18	287	305	305
8	392	400	0	0	0	400
12	174	186	0	0	0	186
0	0	0	12	164	176	176
2	102	104	0	0	0	104
0	0	0	0	98	98	98
1	65	66	0	0	0	66
4	300	304	0	0	0	304
62	763	825	0	0	0	825
4	259	263	0	0	0	263
12	352	364	0	0	0	364

10	372	382	0	0	0	382
0	20	20	0	0	0	20
5	268	273	0	0	0	273
0	0	0	17	111	128	128
0	0	0	44	1330	1374	1374
14	376	390	0	0	0	390
0	0	0	2	210	210	212
0	0	0	0	248	248	248
0	0	0	0	39	39	39
0	0	0	0	79	79	79
7	366	373	0	0	0	373
0	0	0	24	309	333	333
0	0	0	1	101	102	102
0	0	0	2	97	99	99
86	1260	1346	0	0	0	1346
0	240	240	0	0	0	240
0	15	15	0	0	0	15
7	349	356	0	0	0	356
0	0	0	32	230	262	262

4	118	122	0	0	0	122
4	83	87	0	0	0	87
0	36	36	0	0	0	36
0	0	0	8	76	84	84
0	0	0	17	419	436	436
10	333	343	0	0	0	343
0	0	0	14	538	552	552
0	0	0	8	127	135	135
0	0	0	0	20	20	20
0	0	0	0	360	360	360
5	55	60	0	0	0	76
3	12	15	0	45	45	60
0	0	0	11	286	297	297
0	25	25	0	0	0	25
0	0	0	0	16	16	16
0	0	0	17	201	218	218
0	0	0	18	121	139	139
0	50	50	0	50	50	100
0	84	84	0	54	54	138

0	0	0	0	32	32	32
0	0	0	7	165	172	172
0	18	18	0	0	0	37
18	782	800	0	0	0	800
0	0	0	0	182	182	182
0	0	0	0	200	200	200
1	207	208	0	0	0	208
49	1224	1273	0	0	0	1273
0	0	0	4	74	78	78
0	0	0	1	63	64	64
0	0	0	6	178	184	184
15	385	400	0	0	0	400
0	0	0	1	193	194	194
11	182	193	0	0	0	193
0	0	0	4	71	75	75
0	0	0	0	28	28	28
0	0	0	1	68	69	69
0	0	0	10	881	891	891
0	0	0	0	30	30	30

0	0	0	1	80	81	81
69	738	807	62	450	512	2635
0	0	0	0	78	0	78
0	0	0	5	141	146	146
83	1485	1568	0	0	0	1568
2	62	64	0	0	0	64
12	157	169	0	0	0	169
0	0	0	7	83	90	90
9	54	63	0	0	0	63
8	167	175	0	0	0	175
0	0	0	10	401	411	411
0	0	0	1	249	250	250
1	183	184	0	0	0	184
19	177	196	0	0	0	196
0	0	0	17	604	621	621
0	0	0	0	55	55	55
0	0	0	0	18	18	18
1	585	586	0	0	0	586
42	2190	2232	0	0	0	2232

0	0	0	16	559	575	575
0	29	29	0	0	0	29
17	756	773	0	0	0	773
6	194	200	0	0	0	200
0	0	0	9	244	253	253
0	0	0	5	95	100	100
0	0	0	4	68	72	72
7	24	31	0	0	0	31
41	1250	1291	0	0	0	1291
372	7620	7992	0	0	0	7992
6	480	486	0	0	0	486
40	433	473	0	0	0	473
0	0	0	6	60	66	66
0	0	0	15	309	324	324
7	127	134	0	0	0	134
10	570	580	0	0	0	580
0	0	0	6	223	229	229
0	0	0	3	107	110	110
3	86	89	0	0	0	89

0	0	0	3	141	144	144
0	0	0	0	70	70	70
0	0	0	11	77	88	88
0	0	0	14	722	736	736
37	8327	8364	13	819	832	9196
0	0	0	26	1050	1076	1076
0	0	0	54	1026	1080	1080
5	34	39	5	99	104	143
0	0	0	15	64	79	79
25	975	1000	0	0	0	1000
0	0	0	14	520	534	534
0	0	0	9	208	217	217
1	46	47	0	0	0	47
3	47	50	0	0	0	50
0	0	0	0	28	0	28
0	0	0	0	41	41	41
0	66	66	0	0	0	66
0	45	45	0	0	0	45
1	17	18	0	0	0	18

0	15	15	0	0	0	15
0	0	0	1	99	100	100
12	198	210	0	0	0	210
0	0	0	0	120	120	120
0	0	0	0	81	81	81
4	176	180	0	0	0	180
0	0	0	1	140	141	141
0	0	0	0	143	143	143
0	0	0	6	134	140	140
0	0	0	1	40	41	41
2	10	12	0	0	0	12
5	78	83	5	102	107	190
0	12	12	0	0	0	12
7	183	190	0	0	0	190
0	0	0	19	373	392	392
0	0	0	17	314	331	331
0	0	0	46	854	918	918
0	0	0	25	972	997	997
74	1823	1897	0	0	0	1897

0	0	0	51	378	429	429
0	0	0	42	210	252	252
0	0	0	2	24	26	26
21	1585	1606	0	0	0	1606
0	0	0	0	208	208	208
0	0	0	0	85	85	85
66	1251	1317	0	0	0	1317
5	759	764	0	0	0	764
5	551	556	0	0	0	556
1	19	20	0	0	0	20
9	147	156	0	0	0	156
22	354	376	0	0	0	376
12	211	223	0	0	0	223
10	313	323	0	0	0	323

overall procedures		
damaged RLN (n)	healthy RLN (n)	RLN damage rate (%)
13	981	1,31%
62	2.438	2,48%
7	452	1,53%
10	858	1,15%
4	78	4,88%
1	167	0,60%
18	287	5,90%
8	392	2,00%
12	174	6,45%
12	164	6,82%
2	102	1,92%
0	98	0,00%
1	65	1,52%
4	300	1,32%
62	763	7,52%
4	259	1,52%
12	352	3,30%

10	372	2,62%
0	20	0,00%
5	268	1,83%
17	111	13,28%
44	1.330	3,20%
14	376	3,59%
2	210	0,94%
0	248	0,00%
0	39	0,00%
0	79	0,00%
7	366	1,88%
24	309	7,21%
1	101	0,98%
2	97	2,02%
86	1.260	6,39%
0	240	0,00%
0	15	0,00%
5	351	1,40%
32	230	12,21%

4	118	3,28%
4	83	4,60%
0	36	0,00%
8	76	9,52%
17	419	3,90%
10	333	2,92%
14	538	2,54%
8	127	5,93%
0	20	0,00%
0	360	0,00%
5	71	6,58%
3	57	5,00%
11	286	3,70%
0	25	0,00%
0	16	0,00%
17	201	7,80%
18	121	12,95%
0	100	0,00%
0	138	0,00%

0	32	0,00%
7	165	4,07%
1	36	2,70%
18	782	2,25%
0	182	0,00%
0	200	0,00%
1	207	0,48%
49	1.224	3,85%
4	74	5,13%
1	63	1,56%
6	178	3,26%
15	385	3,75%
1	193	0,52%
11	182	5,70%
4	71	5,33%
0	28	0,00%
1	68	1,45%
10	881	1,12%
0	30	0,00%

1	80	1,23%
131	2.504	4,97%
0	78	0,00%
5	141	3,42%
83	1.485	5,29%
2	62	3,13%
12	157	7,10%
7	83	7,78%
9	54	14,29%
8	167	4,57%
10	401	2,43%
1	249	0,40%
1	183	0,54%
19	177	9,69%
17	604	2,74%
0	55	0,00%
0	18	0,00%
1	585	0,17%
42	2.190	1,88%

16	559	2,78%
0	29	0,00%
17	756	2,20%
6	194	3,00%
9	244	3,56%
5	95	5,00%
4	68	5,56%
7	24	22,58%
41	1.250	3,18%
372	7.620	4,65%
6	480	1,23%
40	433	8,46%
6	60	9,09%
15	309	4,63%
7	127	5,22%
10	570	1,72%
6	223	2,62%
3	107	2,73%
3	86	3,37%

3	141	2,08%
0	70	0,00%
11	77	12,50%
14	722	1,90%
50	9.146	0,54%
26	1.050	2,42%
54	1.026	5,00%
10	133	6,99%
15	64	18,99%
25	975	2,50%
14	520	2,62%
9	208	4,15%
1	46	2,13%
3	47	6,00%
0	28	0,00%
0	41	0,00%
0	66	0,00%
0	45	0,00%
1	17	5,56%

0	15	0,00%
1	99	1,00%
12	198	5,71%
0	120	0,00%
0	81	0,00%
4	176	2,22%
1	140	0,71%
0	143	0,00%
6	134	4,29%
1	40	2,44%
2	10	16,67%
10	180	5,26%
0	12	0,00%
7	183	3,68%
19	373	4,85%
17	314	5,14%
46	872	5,01%
25	972	2,51%
74	1.823	3,90%

51	378	11,89%
42	210	16,67%
2	24	7,69%
21	1.585	1,31%
0	208	0,00%
0	85	0,00%
66	1.251	5,01%
5	759	0,65%
5	551	0,90%
1	19	5,00%
9	147	5,77%
22	354	5,85%
12	211	5,38%
10	313	3,10%
