# Supplementary material for: Intraoperative Neuromonitoring Does Not Reduce the Risk of Temporary and Definitive Recurrent Laryngeal Nerve Damage during Thyroid Surgery: A Systematic Review and Meta-Analysis of Endoscopic Findings from 73,325 Nerves at Risk
Source: J Pers Med. 2023 Sep 23;13(10):1429. doi: 10.3390/jpm13101429 (PMC10607766; doi:10.3390/jpm13101429)
Supplement: Supplementary file 1 [file jpm-13-01429-s001.zip › supplementary material S3.pdf]

Reference	Title
Barczyński et al, 2016	The Overwhelming Majority but not All Motor Fibers of the Bifid Recurrent Laryngeal Nerve are Located in the Anterior Extralaryngeal Branch
Bellantone et al, 2011	Is the identification of the external branch of the superior laryngeal nerve mandatory in thyroid operation? Results of a prospective randomized study
Cernea et al, 2011	Negative and positive predictive values of nerve monitoring in thyroidectomy
Chavez et al, 2017	Comparative analysis between a bipolar vessel sealing and cutting device and the tie and suture technique in thyroidectomy: A randomized clinical trial
Chiang et al, 2015	Stimulating dissecting instruments during neuromonitoring of RLN in thyroid surgery
Clayman et al, 2022	Human Amnion/Chorion Membrane May Reduce Transient Recurrent Laryngeal Nerve Injury During Thyroid Surgery
De la Quintana Basarrate et al, 2018	Continuous monitoring of the recurrent laryngeal nerve
De Miguel et al, 2017	Accuracy of transcutaneous laryngeal ultrasound for detecting vocal cord paralysis in the immediate postoperative period after total thyroidectomy
de Pedro Netto et al, 2006	Voice and vocal self-assessment after thyroidectomy
Dionigi et al, 2008a	Defining the learning curve for video-assisted thyroidectomy
Dionigi et al, 2008b	The use of electrothermal bipolar vessel sealing system in minimally invasive video-assisted thyroidectomy (MIVAT)
Dionigi et al, 2008c	Video-assisted thyroidectomy performed in a one-day surgery setting
Dionigi et al, 2008d	What is the learning curve for intraoperative neuromonitoring in thyroid surgery?
Dionigi et al, 2009	Postoperative laryngoscopy in thyroid surgery: proper timing to detect recurrent laryngeal nerve injury
Dionigi et al, 2010	Surgical anatomy and neurophysiology of the vagus nerve (VN) for standardised intraoperative neuromonitoring (IONM) of the inferior laryngeal nerve (ILN) during thyroidectomy
Dionigi et al, 2012	The safety of energy-based devices in open thyroidectomy: A prospective, randomised study comparing the LigaSure™ (LF1212) and the Harmonic® FOCUS
Dionigi et al, 2013	Parathyroid function after open thyroidectomy: A prospective randomized study for ligasure precise versus harmonic FOCUS
Dionigi et al, 2016	Transoral endoscopic thyroidectomy: preliminary experience in Italy
Donnellan et al, 2009	Intraoperative laryngeal nerve monitoring during thyroidectomy
Elsheikh et al, 2016	Voice Changes after Late Recurrent Laryngeal Nerve Identification Thyroidectomy
Enomoto et al, 2014	Recurrent laryngeal nerve palsy during surgery for benign thyroid diseases: risk factors and outcome analysis
Farizon et al, 2008	Intraoperative monitoring of the recurrent laryngeal nerve by vagal nerve stimulation in thyroid surgery
Fík et al, 2014	Minimally invasive video-assisted versus minimally invasive nonendoscopic thyroidectomy
Fregoli et al, 2017	Postoperative Pain Evaluation after Robotic Transaxillary Thyroidectomy Versus Conventional Thyroidectomy: A Prospective Study
Fu et al, 2022	The feasibility of laryngeal nerve protection during thyroidectomy using sternocleidomastoid intermuscular approach with intraoperative neuromonitoring: a case series and step-by-step description of surgical procedure

Gumus et al, 2020	Objective analysis of swallowing and functional voice outcomes after thyroidectomy: A prospective cohort study
Gunes et al, 2019	Effect of intraoperative neuromonitoring on efficacy and safety using sugammadex in thyroid surgery: Randomized clinical trial
Hammad et al, 2016	A Prospective Study Comparing the Efficacy and Surgical Outcomes of Harmonic Focus Scalpel Versus LigaSure Small Jaw in Thyroid and Parathyroid Surgery
Han et al, 2020	Functional Voice and Swallowing Outcome Analysis After Thyroid Lobectomy: Transoral Endoscopic Vestibular Versus Open Approach
Henry et al, 2010	Functional voice outcomes after thyroidectomy: An assessment of the Dsyphonia Severity Index (DSI) after thyroidectomy
Huang et al, 2022	Necessity of Routinely Testing the Proximal and Distal Ends of Exposed Recurrent Laryngeal Nerve During Monitored Thyroidectomy
Hurtado-López et al, 2016	Efficacy of Intraoperative Neuro-Monitoring to Localize the External Branch of the Superior Laryngeal Nerve
Inabnet et al, 2003	Neuromonitoring of the external branch of the superior laryngeal nerve during minimally invasive thyroid surgery under local anesthesia: a prospective study of 10 patients
Iscan et al, 2022	Is craniocaudal dissection of recurrent laryngeal nerve safer than lateral approach: a prospective randomized study comparing both techniques by using continuous intraoperative nerve monitoring
Iyomasa et al, 2019	Laryngeal and vocal alterations after thyroidectomy
Ji et al, 2020	Feasibility and efficacy of intraoperative neural monitoring in remote access robotic and endoscopic thyroidectomy
Ji et al, 2021	Neural Monitoring of the External Branch of the Superior Laryngeal Nerve During Transoral Thyroidectomy
Karaisli et al, 2022	Comparison of stimulating dissector and intermittent stimulating probe for the identification of recurrent laryngeal nerve in reoperative setting
Kletzien et al, 2018	Comparison Between Patient-Perceived Voice Changes and Quantitative Voice Measures in the First Postoperative Year After Thyroidectomy: A Secondary Analysis of a Randomized Clinical Trial
Koçak et al, 1999	Evaluation of vocal cord function after thyroid surgery
Kong et al, 2022	Comparison of a Handheld Device vs Endotracheal Tube-Based Neuromonitoring for Recurrent Laryngeal Nerve Stimulation
Kundra et al, 2010	Laryngoscopic techniques to assess vocal cord mobility following thyroid surgery
Kwon et al, 2015	Role of charcoal tattooing in localization of recurred papillary thyroid carcinoma: Initial experiences
Kwon et al, 2022	Clinical Significance of the Preoperative Thyroidectomy-Related Voice Questionnaire Score in Thyroid Surgery
Lang and Wong, 2011	Feasibility on the use of intraoperative vagal nerve stimulation in gasless, transaxillary endoscopic, and robotic-assisted thyroidectomy
Lang et al, 2011	A comparison of surgical outcomes between endoscopic and robotically assisted thyroidectomy: the authors' initial experience
Lang et al, 2015	Pain and surgical outcomes with and without neck extension in standard open thyroidectomy: A prospective randomized trial
Lavazza et al, 2017	Transoral endoscopic thyroidectomy: Preliminary experience in Italy
Lee et al, 2009	Postauricular and axillary approach endoscopic neck surgery: a new technique
Lee et al, 2010	Outcomes of 109 patients with papillary thyroid carcinoma who underwent robotic total thyroidectomy with central node dissection via the bilateral axillo-breast approach

Lee et al, 2012	Postoperative functional voice changes after conventional open or robotic thyroidectomy: a prospective trial
Lee et al, 2015	The Efficacy of Intraoperative Neuromonitoring During Robotic Thyroidectomy: A Prospective, Randomized Case-Control Evaluation
Li et al, 2021	Advantages of intraoperative nerve monitoring in endoscopic thyroidectomy for papillary thyroid carcinoma
Li et al, 2012	A voice acoustic analysis of thyroid adenoma patients after a unilateral thyroid lobectomy
Li et al, 2022a	Comparison of quality of life and cosmetic result between open and transaxillary endoscopic thyroid lobectomy for papillary thyroid microcarcinoma survivors: A single-center prospective cohort study
Li et al, 2022b	Thyroidectomy using a single-port cervico-mental angle approach
Liang et al, 2022	Thyroidectomy for thyroid cancer via transareola single-site endoscopic approach: results of a case-match study with large-scale population
Lin et al, 2021	Comparative study of gasless endoscopic selective lateral neck dissection via the anterior chest approach versus conventional open surgery for papillary thyroid carcinoma
Liu et al, 2003	Minimally invasive low-collar incision in thyroid lobectomy
Liu et al, 2016	Exclusive real-time monitoring during recurrent laryngeal nerve dissection in conventional monitored thyroidectomy
Liu et al, 2018	Laryngeal nerve morbidity in 1.273 central node dissections for thyroid cancer
Lombardi et al, 2006	Voice and swallowing changes after thyroidectomy in patients without inferior laryngeal nerve injuries
Lombardi et al, 2012	Prospective electromyographic evaluation of functional postthyroidectomy voice and swallowing symptoms.
Lou et al, 2022	Transoral Endoscopic Thyroidectomy Vestibular Approach (TOETVA) in the Perioperative Mode of Day Ward
Mangano et al, 2015	Continuous intraoperative neuromonitoring in thyroid surgery: Safety analysis of 400 consecutive electrode probe placements with standardized procedures
Marchese et al, 2021	Neck complaints before and after uncomplicated thyroidectomy: prevalence, postoperative outcome and relationships with thyroid weight and reflux like symptoms
Mazzone et al, 2021	Continuous Intraoperative Nerve Monitoring in Thyroid Surgery: Can Amplitude Be a Standardized Parameter?
Mehanna et al, 2015	Effect of endotracheal tube size on vocal outcomes after thyroidectomy: A randomized clinical trial
Miccoli et al, 2000	Minimally invasive video-assisted surgery of the thyroid: a preliminary report.
Miccoli et al, 2001	Impact of harmonic scalpel on operative time during video-assisted thyroidectomy
Miccoli et al, 2004	Minimally invasive video-assisted thyroidectomy: five years of experience
Miccoli et al, 2007	Video-assisted central compartment lymphadenectomy in a patient with a positive RET oncogene: initial experience
Miccoli et al, 2020	Minimally invasive video-assisted thyroidectomy
Mishra et al, 2007	The external laryngeal nerve in thyroid surgery: The 'no more neglected' nerve
Mohil et al, 2011	Recurrent laryngeal nerve and voice preservation: routine identification and appropriate assessment - two important steps in thyroid surgery
Moreira et al, 2020	Investigation of recurrent laryngeal palsy rates for potential associations during thyroidectomy

Netto et al, 2007	Vocal fold immobility after thyroidectomy with intraoperative recurrent laryngeal nerve monitoring
Nguyen et al, 2022	Comparison of Transoral Endoscopic Thyroidectomy Vestibular Approach and Conventional Open Thyroidectomy in Benign Thyroid Tumors
Onoda et al, 2019	Continuous intraoperative neuromonitoring for thyroid cancer surgery: A prospective study
Pardal-Refoyo, 2015	Usefulness of neuromonitoring in thyroid surgery
Park et al, 2013	How can we screen voice problems effectively in patients undergoing thyroid surgery?
Park et al, 2015	Robotic thyroidectomy learning curve for beginning surgeons with little or no experience of endoscopic surgery
Park et al, 2018	Long-term voice outcome after thyroidectomy using energy based devices
Périé et al, 2013	Value of recurrent laryngeal nerve monitoring in the operative strategy during total thyroidectomy and parathyroidectomy
Piccoli et al, 2019	Evolution Strategies in Transaxillary Robotic Thyroidectomy: Considerations on the First 449 Cases Performed
Procacciante et al, 2000	Palpatory method used to identify the recurrent laryngeal nerve during thyroidectomy
Puntambekar et al, 2007	Endoscopic thyroidectomy: Our technique
Randolph et al, 2004	Recurrent laryngeal nerve identification and assessment during thyroid surgery: laryngeal palpation
Revelli et al, 2023	Is There Any Reliable Predictor of Functional Recovery Following Post-thyroidectomy Vocal Fold Paralysis?
Roh et al, 2009	Recurrent laryngeal nerve paralysis in patients with papillary thyroid carcinomas: evaluation and management of resulting vocal dysfunction
Rohaizak et al, 2021	Vagus Nerve Injury during Continuous Intraoperative Neuromonitoring (cIONM) for Thyroid Surgery: Assessment of Severity
Russell et al, 2021	Transoral Thyroidectomy: Safety and Outcomes of 200 Consecutive North American Cases
Rybakovas et al, 2019	Recurrent laryngeal nerve injury assessment by intraoperative laryngeal ultrasonography: A prospective diagnostic test accuracy study
Saavedra-Perez et al, 2022	Thyroidectomy via unilateral axillo-breast approach (UABA) with gas insufflation: prospective multicentre European study
Santosh et al, 2014	Capsular dissection method in thyroidectomy
Schardey et al, 2010	Invisible scar endoscopic dorsal approach thyroidectomy: a clinical feasibility study.
Schneider et al, 2016	Dynamics of loss and recovery of the nerve monitoring signal during thyroidectomy predict early postoperative vocal fold function
Schneider et al, 2019	Complete and incomplete recurrent laryngeal nerve injury after thyroid and parathyroid surgery: Characterizing paralysis and paresis
Schneider et al, 2021	Recurrent laryngeal nerve preservation strategies in pediatric thyroid oncology: Continuous vs. intermittent nerve monitoring
Senosiain et al, 2022	Utility of the continuous intraoperative neuromonitoring in the prevention of the recurrent laryngeal nerve paralysis during thyroid surgery. A prospective observational study
Sheahan et al, 2012	Risk factors for recurrent laryngeal nerve neuropraxia postthyroidectomy
Sinclair et al, 2018	Noninvasive, tube-based, continuous vagal nerve monitoring using the laryngeal adductor reflex: Feasibility study of 134 nerves at risk

Sitges-Serra et al, 2013	Prospective study on loss of signal on the first side during neuromonitoring of the recurrent laryngeal nerve in total thyroidectomy
Song et al, 2019	Comparison of postoperative voice outcomes after postauricular facelift robotic hemithyroidectomy and conventional transcervical hemithyroidectomy
Song et al, 2020	Voice outcomes of transoral robotic thyroidectomy: Comparison with conventional trans-cervical thyroidectomy
Song et al, 2021	Health-related quality of life after transoral robotic thyroidectomy in papillary thyroid carcinoma
Souza et al, 2009	Laryngeal vocal and endoscopic alterations after thyroidectomy under local anesthesia and hypnosedation
Sreejayan et al, 2019	Inferior Approach: a Safe Method for Identification of Recurrent Laryngeal Nerve During Thyroidectomy
Staubitz et al, 2020	Effect of intraoperative nerve monitoring on postoperative vocal cord palsy rates after thyroidectomy: European multicentre registry-based study
Steurer et al, 2003	Functional Laryngeal Results after Thyroidectomy and Extensive Recurrent Laryngeal Nerve Dissection Without Neuromonitoring - An Analysis of More Than 1 000 Nerves at Risk
Steurer, 2002	Advantages of recurrent laryngeal nerve identification in thyroidectomy and parathyroidectomy and the importance of preoperative and postoperative laryngoscopic examination in more than 1000 nerves at risk
Stevens et al, 2012	The impact of recurrent laryngeal neuromonitoring on multi-dimensional voice outcomes following thyroid surgery
Stojadinovic et al, 2002	Prospective functional voice assessment in patients undergoing thyroid surgery
Stopa and Barczyński, 2017	Prognostic value of intraoperative neural monitoring of the recurrent laryngeal nerve in thyroid surgery
Tae et al, 2012a	Early surgical outcomes of robotic thyroidectomy by a gasless unilateral axillo-breast or axillary approach for papillary thyroid carcinoma: 2 years' experience
Tae et al, 2012b	Functional voice and swallowing outcomes after robotic thyroidectomy by a gasless unilateral axillo-breast approach: comparison with open thyroidectomy
Tae et al, 2019	Early experience of transoral thyroidectomy: Comparison of robotic and endoscopic procedures
Taylor et al, 2020	Changes in Tracheal Tube Cuff Pressure and Recurrent Laryngeal Nerve Conductivity During Thyroid Surgery
Teitelbaum and Wenig, 1995	Superior laryngeal nerve injury from thyroid surgery
Terris and Chin, 2006	Clinical implementation of endoscopic thyroidectomy in selected patients
Terris et al, 2006	Ultrasonic technology facilitates minimal access thyroid surgery
Terris et al, 2010	Reoperative thyroidectomy for benign thyroid disease
Terris et al, 2011	Robotic facelift thyroidectomy: II. Clinical feasibility and safety
Timon et al, 2010	Investigation of the impact of thyroid surgery on vocal tract steadiness
Tiwari et al, 2018	Prospective study of complications following surgery for thyroid malignancy: A tertiary cancer care centre experience
Uludag et al, 2016	Contribution of intraoperative neural monitoring to preservation of the external branch of the superior laryngeal nerve: a randomized prospective clinical trial
Uludag et al, 2017	Effect of energy-based devices on voice quality after total thyroidectomy
Van Lierde et al, 2010	Impact of thyroidectomy without laryngeal nerve injury on vocal quality characteristics: an objective multiparameter approach
Van Slycke et al, 2013	Initial experience with S-shaped electrode for continuous vagal nerve stimulation in thyroid surgery

Vaysberg and Steward, 2008	Minimally invasive video-assisted thyroidectomy
Vicente et al, 2014	Voice outcomes after total thyroidectomy, partial thyroidectomy, or non-neck surgery using a prospective multifactorial assessment
Wasserman et al, 2008	Determination of the function of the internal branch of the superior laryngeal nerve after thyroidectomy
Wilhelm et al, 2011	Endoscopic minimally invasive thyroidectomy (eMIT): a prospective proof-of-concept study in humans.
Witt et al, 2005	Recurrent laryngeal nerve electrophysiologic monitoring in thyroid surgery: The standard of care?
Witzel, 2007	The axillary access in unilateral thyroid resection
Wojtczak et al, 2018	Evaluating the introduction of intraoperative neuromonitoring of the recurrent laryngeal nerve in thyroid and parathyroid surgery
Wolff et al, 2022	Factors Associated With Injury to Recurrent Laryngeal Nerve in Patients Undergoing Surgery for Thyroid Cancer: A Single-centre Study Using Translaryngeal Ultrasound
Wong et al, 2015	Assessing the Validity of Transcutaneous Laryngeal Ultrasonography (TLUSG) After Thyroidectomy: What Factors Matter?
Wong et al, 2019	Vocal Cord Palsies Missed by Transcutaneous Laryngeal Ultrasound (TLUSG): Do They Experience Worse Outcomes?
Wu et al, 2013	Video-assisted selective lateral neck dissection for papillary thyroid carcinoma
Wu et al, 2018	Staged Thyroidectomy: A Single Institution Perspective
Yildirim et al, 2008	Ultrasonic harmonic scalpel in total thyroidectomies
Yilmaz et al, 2018	An analysis on aerodynamic and acoustic changes after thyroidectomy
Yu et al, 2022	Effects of Intraoperative Neural Tunnel in Protecting Recurrent Laryngeal Nerve: Experiences in Open, Trans Breast, and Transoral Endoscopic Thyroidectomy
Yuan et al, 2022a	Total thyroidectomy versus hemithyroidectomy with intraoperative radiofrequency ablation for unilateral thyroid cancer with contralateral nodules: A propensity score matching study
Yuan et al, 2022b	Visual identification and neuromonitoring vs. no sighting the external branch of the superior laryngeal nerve in thyroid surgery: a randomized clinical trial
Zavdy et al, 2021	Intraoperative Ultrasonographic Assessment of Vocal Cord motion under sedation, following paediatric thyroidectomy in the Era of COVID-19: A double-blinded preliminary study
Zhang et al, 2017	Percutaneous probe stimulation for intraoperative neuromonitoring in total endoscopic thyroidectomy: A preliminary experience
Zhang et al, 2019	Central Lymph Node Dissection by Endoscopic Bilateral Areola Versus Open Thyroidectomy
Zhang et al, 2021	Drawbacks of neural monitoring troubleshooting algorithms in transoral endoscopic thyroidectomy
Zhang et al, 2022	Clinical Experience of Use of Percutaneous Continuous Nervemonitoring in Robotic Bilateral Axillo-Breast Thyroid Surgery

Procedures included (n)	procedures with IONM		procedures without IONM		overall pr
	RLN at risk	Irreversible damages	RLN at risk	Irreversible damages	RLN at risk
1268	2500	10	0	0	2500
289	0	0	459	2	459
447	868	2	0	0	868
41	82	0	0	0	82
100	168	0	0	0	168
201	0	0	305	0	305
248	400	0	0	0	400
93	186	3	0	0	186
88	0	0	176	5	176
67	104	0	0	0	104
63	0	0	98	0	98
43	66	1	0	0	66
152	304	0	0	0	304
434	825	6	0	0	825
140	263	0	0	0	263
182	364	0	0	0	364
199	382	0	0	0	382
15	20	0	0	0	20
210	273	0	0	0	273
64	0	0	128	0	128
844	0	0	1374	10	1374
195	390	2	0	0	390
131	0	0	210	0	212
124	0	0	248	0	248
39	0	0	39	0	39

40	0	0	79	0	79
203	373	0	0	0	373
294	0	0	333	1	333
102	0	0	102	0	102
62	0	0	99	2	99
796	1346	2	0	0	1346
148	240	0	0	0	240
10	15	0	0	0	15
198	356	2	0	0	356
151	0	0	262	5	262
104	122	0	0	0	122
76	87	1	0	0	87
32	36	0	0	0	36
42	0	0	84	0	84
218	0	0	436	0	436
249	343	2	0	0	343
104	0	0	135	0	135
10	0	0	20	0	20
180	0	0	360	0	360
60	60	1	0	0	76
46	15	1	45	0	60
180	0	0	297	2	297
15	25	0	0	0	25
10	0	0	16	0	16
109	0	0	218	1	218

88	0	0	139	0	139
50	50	0	50	0	100
69	84	0	54	0	138
32	0	0	32	0	32
172	0	0	172	0	172
30	18	0	0	0	37
400	800	5	0	0	800
91	0	0	182	0	182
200	0	0	200	0	200
120	208	0	0	0	208
1119	1273	4	0	0	1273
39	0	0	78	0	78
32	0	0	64	0	64
157	0	0	184	0	184
211	400	0	0	0	400
97	0	0	194	0	194
108	193	4	0	0	193
49	0	0	75	0	75
27	0	0	28	0	28
116	0	0	69	0	69
579	0	0	891	8	891
15	0	0	30	0	30
67	0	0	81	0	81
46	0	0	0	0	78
100	0	0	146	0	146
1003	1568	1	0	0	1568

104	169	6	0	0	169
78	0	0	90	0	90
43	63	0	0	0	63
91	175	0	0	0	175
242	0	0	411	5	411
125	0	0	250	0	250
92	184	1	0	0	184
100	196	4	0	0	196
449	0	0	621	1	621
45	0	0	55	0	55
15	0	0	18	0	18
449	586	1	0	0	586
51	2232	9	0	0	2232
319	0	0	575	5	575
18	29	0	0	0	29
533	773	1	0	0	773
112	200	0	0	0	200
253	0	0	253	0	253
80	0	0	100	0	100
30	31	1	0	0	31
785	1291	24	0	0	1291
4707	7992	36	0	0	7992
258	486	1	0	0	486
248	473	3	0	0	473
209	0	0	324	1	324
100	134	2	0	0	134

290	580	1	0	0	580
110	0	0	110	0	110
89	89	0	0	0	89
114	0	0	144	0	144
35	0	0	70	10	70
393	0	0	736	0	736
4598	8364	4	832	2	9196
624	0	0	1076	3	1076
608	0	0	1080	6	1080
91	39	1	104	2	143
50	0	0	79	0	79
500	1000	5	0	0	1000
301	0	0	14	1	534
111	0	0	217	1	217
37	47	0	0	0	47
32	50	1	0	0	50
20	0	0	0	0	28
36	0	0	41	0	41
44	66	0	0	0	66
45	45	0	0	0	45
18	18	0	0	0	18
10	15	0	0	0	15
50	0	0	100	0	100
133	210	1	0	0	210
60	0	0	120	0	120
44	0	0	81	0	81
100	180	1	0	0	180

86	0	0	141	0	141
112	0	0	143	0	143
33	0	0	41	0	41
8	12	0	0	0	12
136	83	1	107	2	190
12	12	0	0	0	12
101	190	2	0	0	190
196	0	0	392	4	392
581	0	0	918	7	918
1196	1897	14	0	0	1897
26	0	0	26	0	26
803	1606	2	0	0	1606
104	0	0	208	0	208
44	0	0	85	0	85
929	1317	0	0	0	1317
382	764	1	0	0	764
278	556	0	0	0	556
15	20	0	0	0	20
132	156	0	0	0	156
400	376	0	0	0	376
179	223	1	0	0	223
304	323	0	0	0	323

rocedures
Irreversible damages
10
2
2
0
0
0
0
3
5
0
0
1
0
6
0
0
0
0
0
0
10
2
0
0
0

0
0
1
0
2
2
0
0
2
5
0
1
0
0
0
2
0
0
0
1
1
2
0
0
1

0
0
0
0
0
0
5
0
0
0
4
0
0
0
0
0
4
0
0
0
8
0
0
0
0
1

6
0
0
0
5
0
1
4
1
0
0
1
9
5
0
1
0
0
0
1
24
36
1
3
1
2

1
0
0
0
10
0
6
3
6
3
0
5
1
1
0
1
0
0
0
0
0
0
0
1
0
0
1

0
0
0
0
3
0
2
4
7
14
0
2
0
0
0
1
0
0
0
0
1
0
