# Supplementary material for: Intraoperative Neuromonitoring Does Not Reduce the Risk of Temporary and Definitive Recurrent Laryngeal Nerve Damage during Thyroid Surgery: A Systematic Review and Meta-Analysis of Endoscopic Findings from 73,325 Nerves at Risk
Source: J Pers Med. 2023 Sep 23;13(10):1429. doi: 10.3390/jpm13101429 (PMC10607766; doi:10.3390/jpm13101429)
Supplement: Supplementary file 1 [file jpm-13-01429-s001.zip › supplementary material S4.pdf]

Reference	I-IONM			
	RLN at risk (n)	Temporary damaged RLN (n)	Irreversibly damaged RLN (n)	RLN at risk (n)
Barczyński et al, 2016	2500	52	10	0
Cernea et al, 2011	868	8	2	0
Chavez et al, 2017	0	0	0	82
Chiang et al, 2015	168	1	0	0
De la Quintana Basarrate et al, 2018	0	0	0	400
De Miguel et al, 2017	186	9	3	0
Dionigi et al, 2008a	104	2	0	0
Dionigi et al, 2008c	66	1	0	0
Dionigi et al, 2008d	304	4	0	0
Dionigi et al, 2010	263	4	0	0
Dionigi et al, 2016	20	0	0	0
Donnellan et al, 2009	273	5	0	0
Fu et al, 2022	39	0	0	0
Gunes et al, 2019	0	0	0	373
Huang et al, 2022	1346	84	2	0
Hurtado-López et al, 2016	240	0	0	0
Iscan et al, 2022	0	0	0	356
Ji et al, 2020	122	4	0	0
Ji et al, 2021	87	3	1	0
Karaisli et al, 2022	36	0	0	0
Kong et al, 2022	343	8	2	0
Lavazza et al, 2017	25	0	0	0
Lee et al, 2015	100	0	0	0
Li et al, 2021	138	0	0	0
Li et al, 2022b	37	0	0	0
Liu et al, 2018	1273	45	4	0
Mangano et al, 2015	0	0	0	400
Mazzone et al, 2021	0	0	0	193
Mirallie et al, 2018	2635	63	6	0
Moreira et al, 2020	0	0	0	1568
Netto et al, 2007	169	6	6	0
Onoda et al, 2019	0	0	0	63
Pardal-Refoyo, 2015	175	8	0	0
Périé et al, 2013	196	15	4	0
Randolph et al, 2004	0	0	0	586
Revelli et al, 2023	2232	33	9	0
Rohaizak et al, 2021	1545	74	17	777
Russell et al, 2021	773	16	1	0
Rybakovas et al, 2019	200	6	0	0
Schardey et al, 2010	31	6	1	0
Schneider et al, 2016	0	0	0	1291
Schneider et al, 2019	7992	336	36	0
Schneider et al, 2021	404	5	1	82
Senosiain et al, 2022	0	0	0	473
Sinclair et al, 2018	0	0	0	134
Sitges-Serra et al, 2013	580	9	1	0
Song et al, 2020	89	3	0	0
Staubitz et al, 2020	9196	33	4	0
Stopa and Barczyński, 2017	1000	20	5	0
Taylor et al, 2020	0	0	0	50
Terris et al, 2010	45	0	0	0
Timon et al, 2010	15	0	0	0
Van Slycke et al, 2013	0	0	0	180
Wilhelm et al, 2011	12	2	0	0
Witt et al, 2005	190	3	2	0

Witzel, 2007	12	0	0	0
Wojtczak et al, 2018	190	5	2	0
Wong et al, 2019	1897	60	14	0
Wu et al, 2018	1606	19	2	0
Yuan et al, 2022a	764	4	1	0
Zavdy et al, 2021	20	1	0	0
Zhang et al, 2017	156	9	0	0
Zhang et al, 2019	376	22	0	0
Zhang et al, 2021	223	12	1	0
Zhang et al, 2022	0	0	0	323

C-IONM		Overall		
Temporary damaged RLN (n)	*Irreversibly damaged RLN (n)*	*RLN at risk (n)*	*Temporary damaged RLN (n)*	*Irreversibly damaged RLN (n)*
0	0	2500	62	10
0	0	868	10	2
4	0	82	4	0
0	0	168	1	0
8	0	400	8	0
0	0	186	12	3
0	0	104	2	0
0	0	66	1	1
0	0	304	4	0
0	0	263	4	0
0	0	20	0	0
0	0	273	5	0
0	0	39	0	0
7	0	373	7	0
0	0	1346	86	2
0	0	240	0	0
5	2	356	5	2
0	0	122	4	0
0	0	87	4	1
0	0	36	0	0
0	0	343	10	2
0	0	25	0	0
0	0	100	0	0
0	0	138	0	0
0	0	37	1	0
0	0	1273	49	4
15	0	400	15	0
7	4	193	11	4
0	0	2635	131	12
83	1	1568	83	1
0	0	169	12	6
9	0	63	9	0
0	0	175	8	0
0	0	196	19	4
1	1	586	1	1
0	0	2232	42	9
20	0	2869	141	29
0	0	773	17	1
0	0	200	6	0
0	0	31	7	1
41	24	1291	41	24
0	0	7992	372	36
0	0	486	6	1
37	3	473	40	3
5	2	134	7	2
0	0	580	10	1
0	0	89	3	0
0	0	9196	50	6
0	0	1000	25	5
2	1	50	3	1
0	0	45	0	0
0	0	15	0	0
3	1	180	4	1
0	0	12	2	0
0	0	190	10	3

0	0	12	0	0
0	0	190	7	2
0	0	1897	74	14
0	0	1606	21	2
0	0	764	5	1
0	0	20	1	0
0	0	156	9	0
0	0	376	22	0
0	0	223	12	1
10		323	10	0
