# Supplementary material for: Intraoperative Neuromonitoring Does Not Reduce the Risk of Temporary and Definitive Recurrent Laryngeal Nerve Damage during Thyroid Surgery: A Systematic Review and Meta-Analysis of Endoscopic Findings from 73,325 Nerves at Risk
Source: J Pers Med. 2023 Sep 23;13(10):1429. doi: 10.3390/jpm13101429 (PMC10607766; doi:10.3390/jpm13101429)
Supplement: Supplementary file 1 [file jpm-13-01429-s001.zip › Supplementary material S8.pdf]

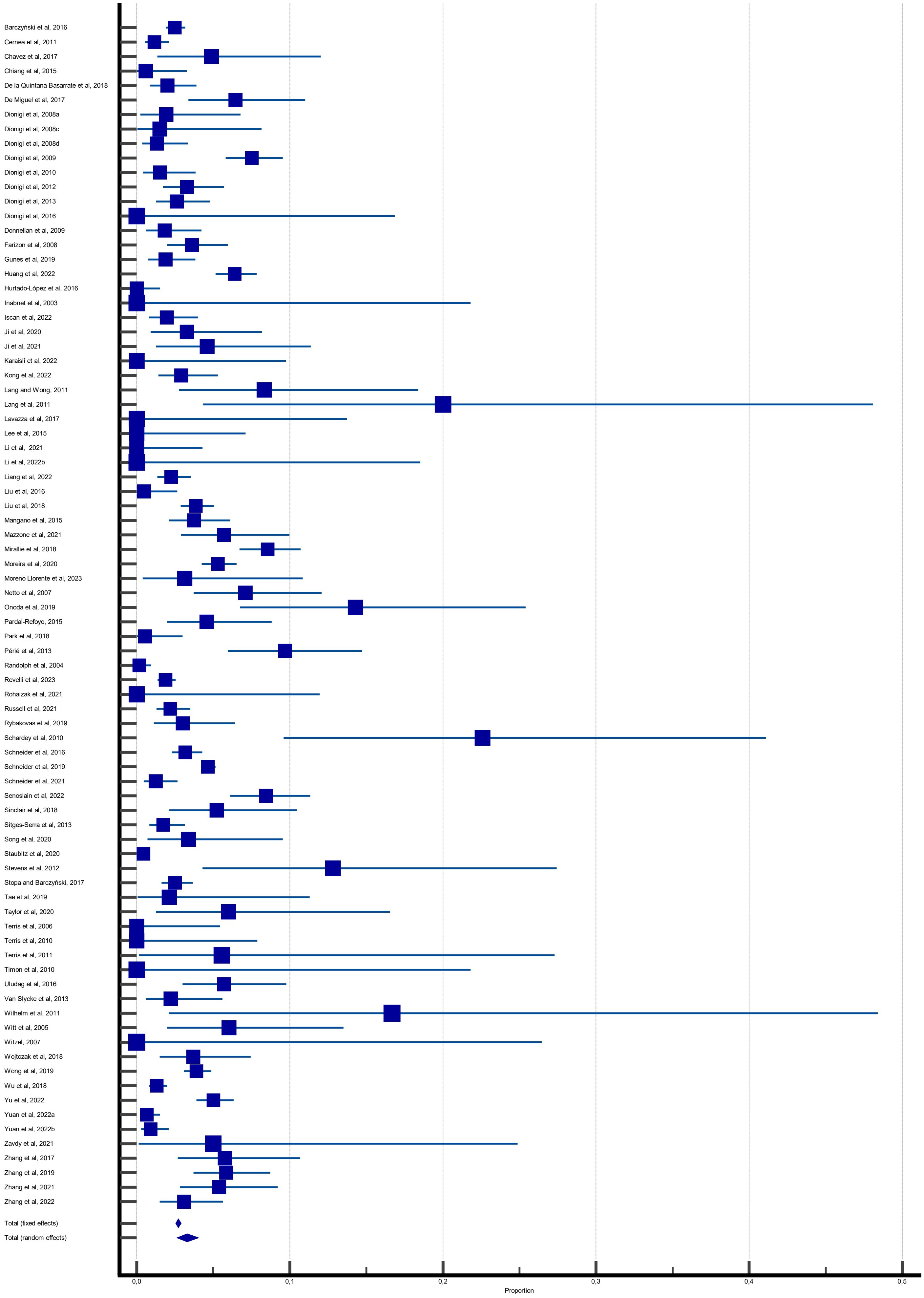
Barczyński et al, 2016
Cernea et al, 2011
Chavez et al, 2017
Chiang et al, 2015
De la Quintana Basarrate et al, 2018
De Miguel et al, 2017
Dionigi et al, 2008a
Dionigi et al, 2008c
Dionigi et al, 2008d
Dionigi et al, 2009
Dionigi et al, 2010
Dionigi et al, 2012
Dionigi et al, 2013
Dionigi et al, 2016
Donnellan et al, 2009
Farizon et al, 2008
Gunes et al, 2019
Huang et al, 2022
Hurtado-López et al, 2016
Inabnet et al, 2003
Iscan et al, 2022
Ji et al, 2020
Ji et al, 2021
Karaisli et al, 2022
Kong et al, 2022
Lang and Wong, 2011
Lang et al, 2011
Lavazza et al, 2017
Lee et al, 2015
Li et al, 2021
Li et al, 2022b
Liang et al, 2022
Liu et al, 2016
Liu et al, 2018
Mangano et al, 2015
Mazzone et al, 2021
Mirallie et al, 2018
Moreira et al, 2020
Moreno Llorente et al, 2023
Netto et al, 2007
Onoda et al, 2019
Pardal-Refoyo, 2015
Park et al, 2018
Périé et al, 2013
Randolph et al, 2004
Revelli et al, 2023
Rohaizak et al, 2021
Russell et al, 2021
Rybakovas et al, 2019
Schardey et al, 2010
Schneider et al, 2016
Schneider et al, 2019
Schneider et al, 2021
Senosiain et al, 2022
Sinclair et al, 2018
Sitges-Serra et al, 2013
Song et al, 2020
Staubitz et al, 2020
Stevens et al, 2012
Stopa and Barczyński, 2017
Tae et al, 2019
Taylor et al, 2020
Terris et al, 2006
Terris et al, 2010
Terris et al, 2011
Timon et al, 2010
Uludag et al, 2016
Van Slycke et al, 2013
Wilhelm et al, 2011
Witt et al, 2005
Witzel, 2007
Wojtczak et al, 2018
Wong et al, 2019
Wu et al, 2018
Yu et al, 2022
Yuan et al, 2022a
Yuan et al, 2022b
Zavdy et al, 2021
Zhang et al, 2017
Zhang et al, 2019
Zhang et al, 2021
Zhang et al, 2022
Total (fixed effects)
Total (random effects)
0,0
0,1
0,2
0,3
0,4
0,5
Proportion
