# Supplementary figures and images for: Intraoperative Neuromonitoring Does Not Reduce the Risk of Temporary and Definitive Recurrent Laryngeal Nerve Damage during Thyroid Surgery: A Systematic Review and Meta-Analysis of Endoscopic Findings from 73,325 Nerves at Risk
Source: J Pers Med. 2023 Sep 23;13(10):1429. doi: 10.3390/jpm13101429 (PMC10607766; doi:10.3390/jpm13101429)

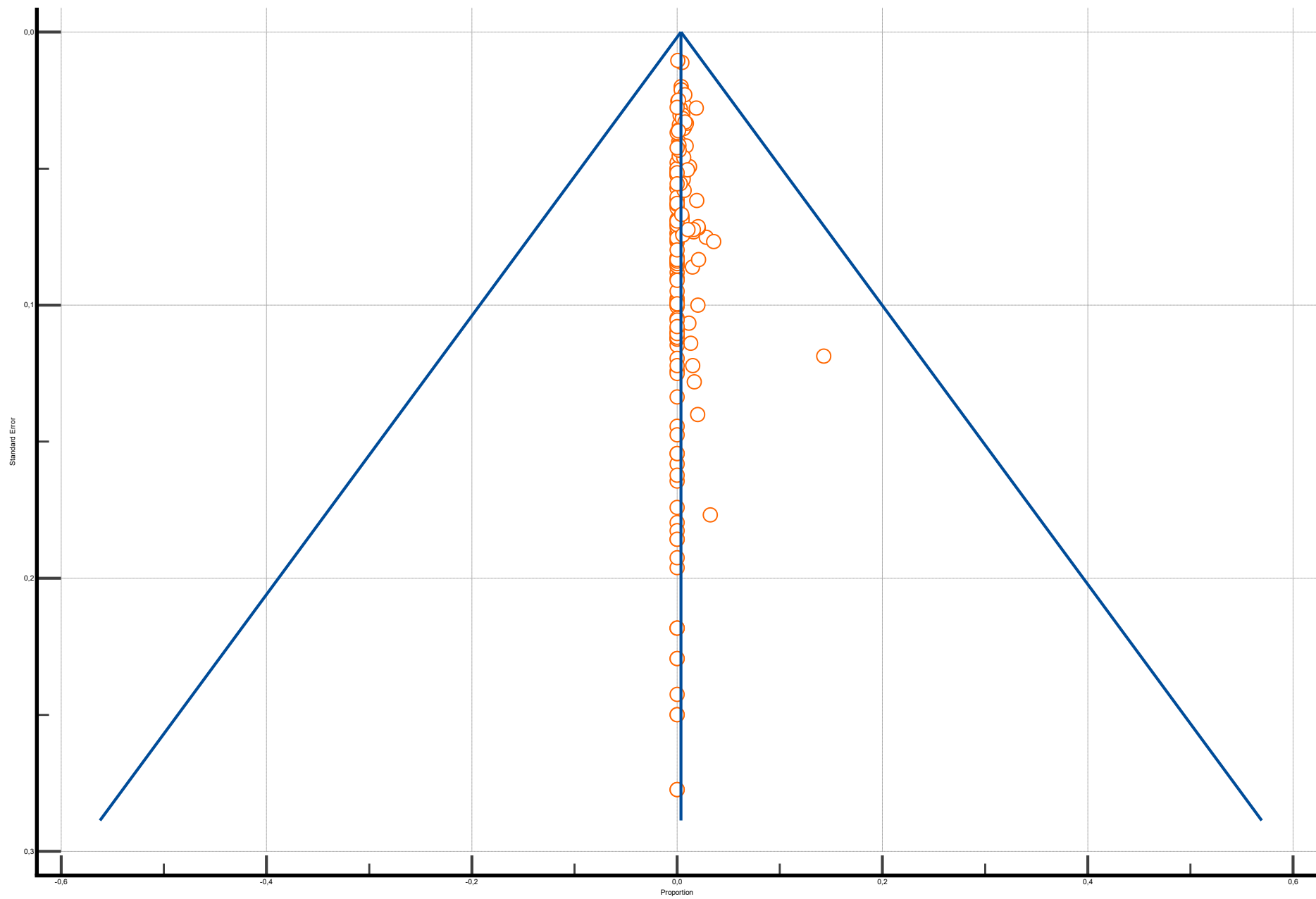

Supplement: Supplementary file 1 [file jpm-13-01429-s001.zip › Supplementary material S11.pdf]

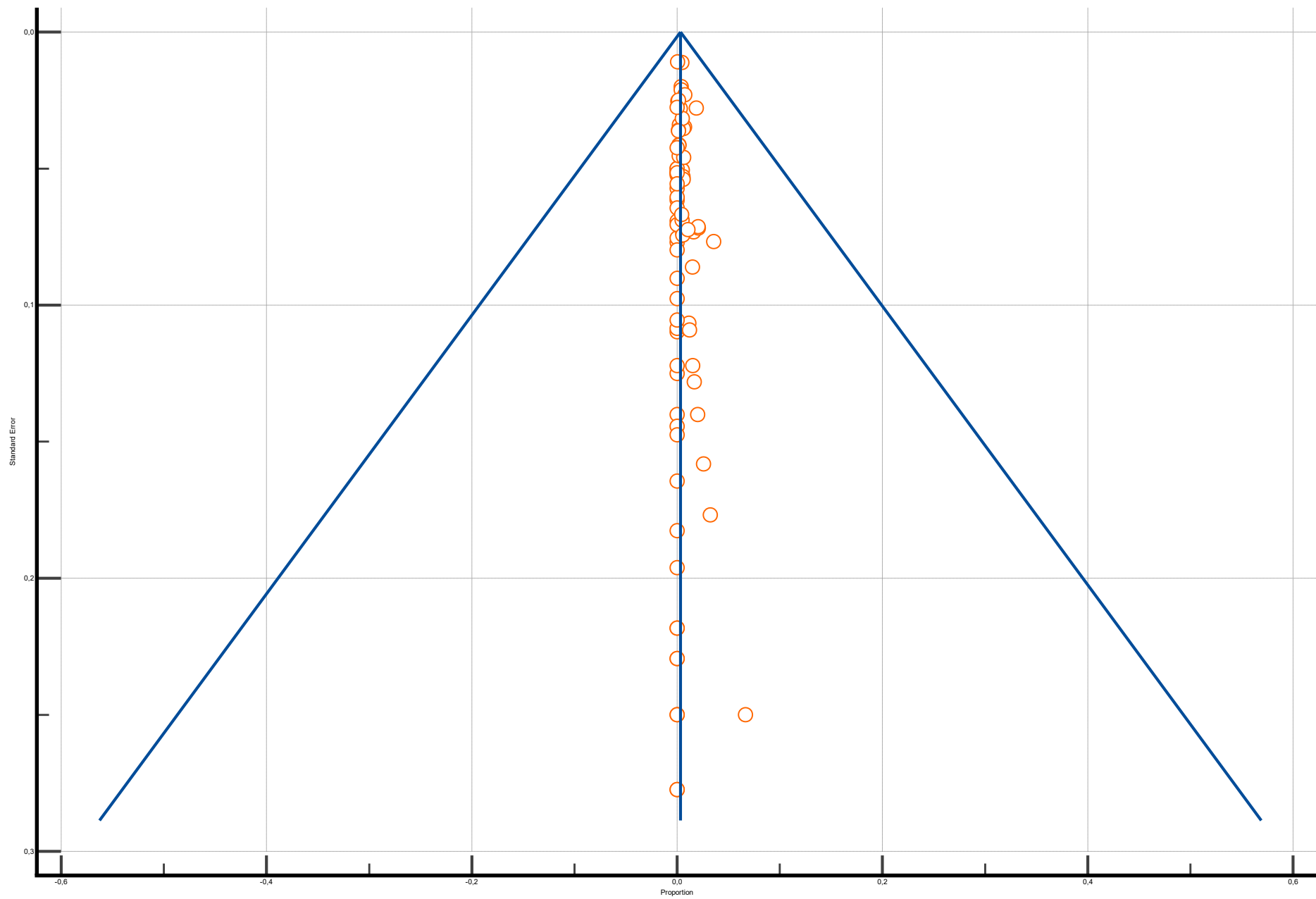

Supplement: Supplementary file 1 [file jpm-13-01429-s001.zip › Supplementary material S13.pdf]

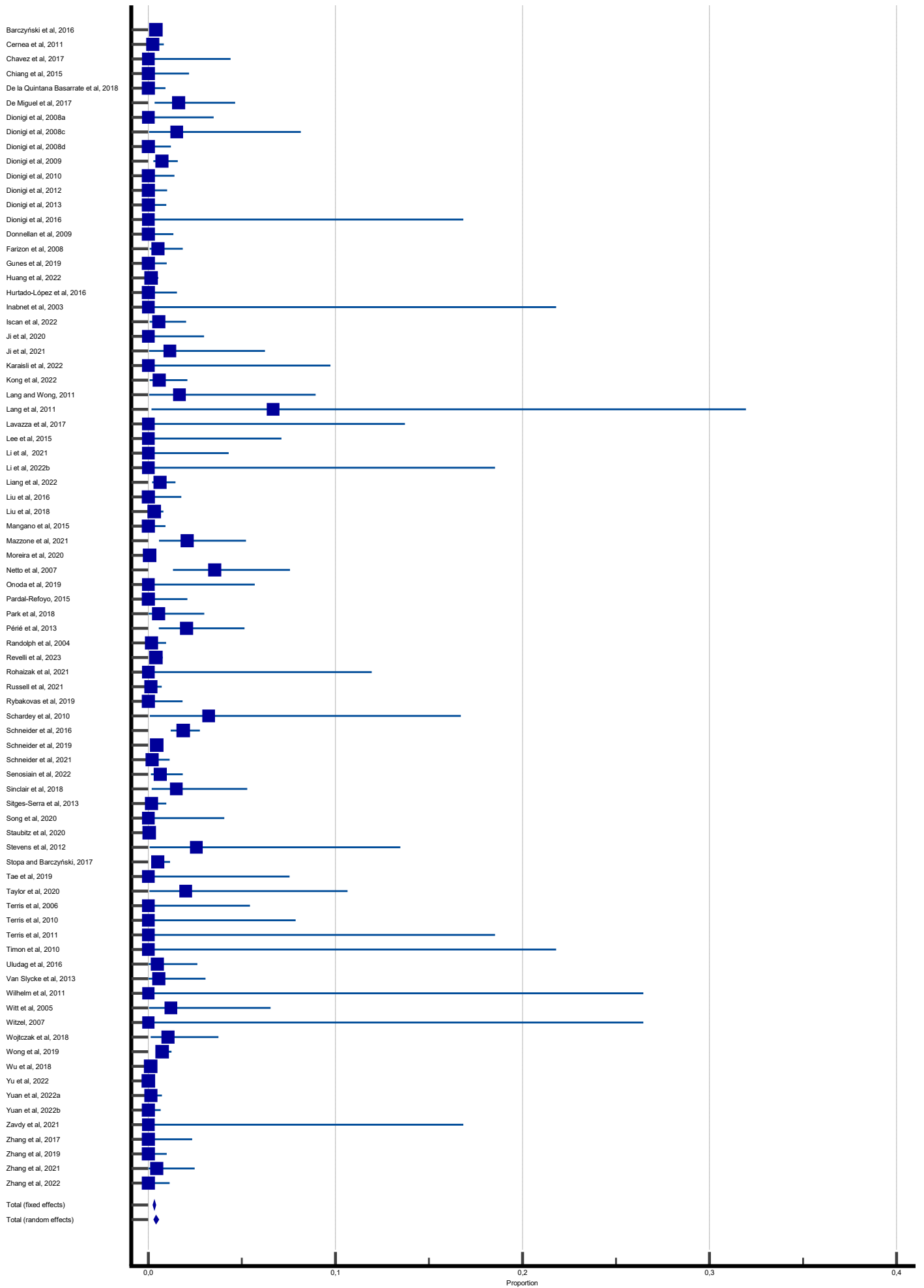

Supplement: Supplementary file 1 [file jpm-13-01429-s001.zip › Supplementary material S14.pdf]

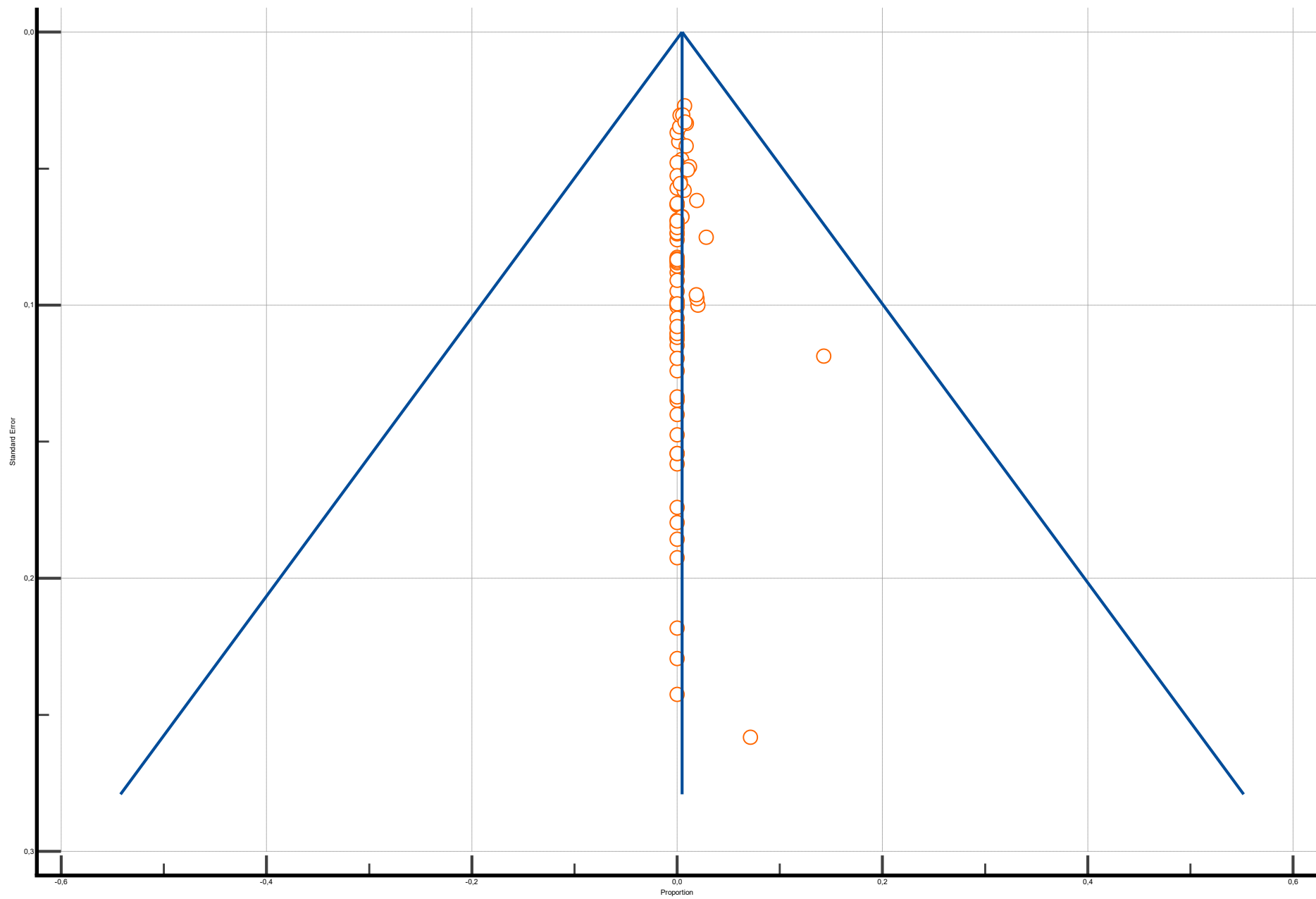

Supplement: Supplementary file 1 [file jpm-13-01429-s001.zip › Supplementary material S15.pdf]

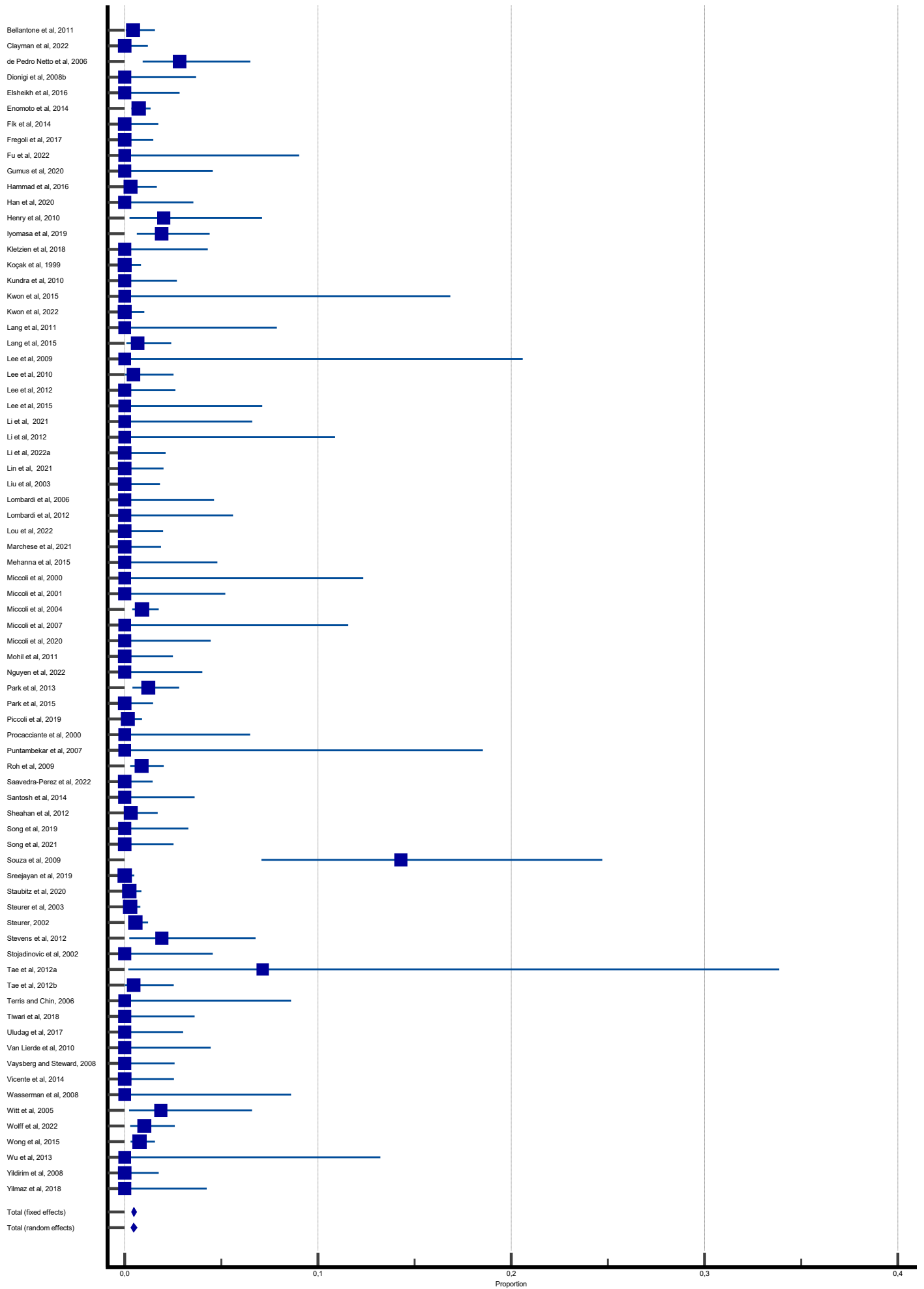

Supplement: Supplementary file 1 [file jpm-13-01429-s001.zip › Supplementary material S16.pdf]

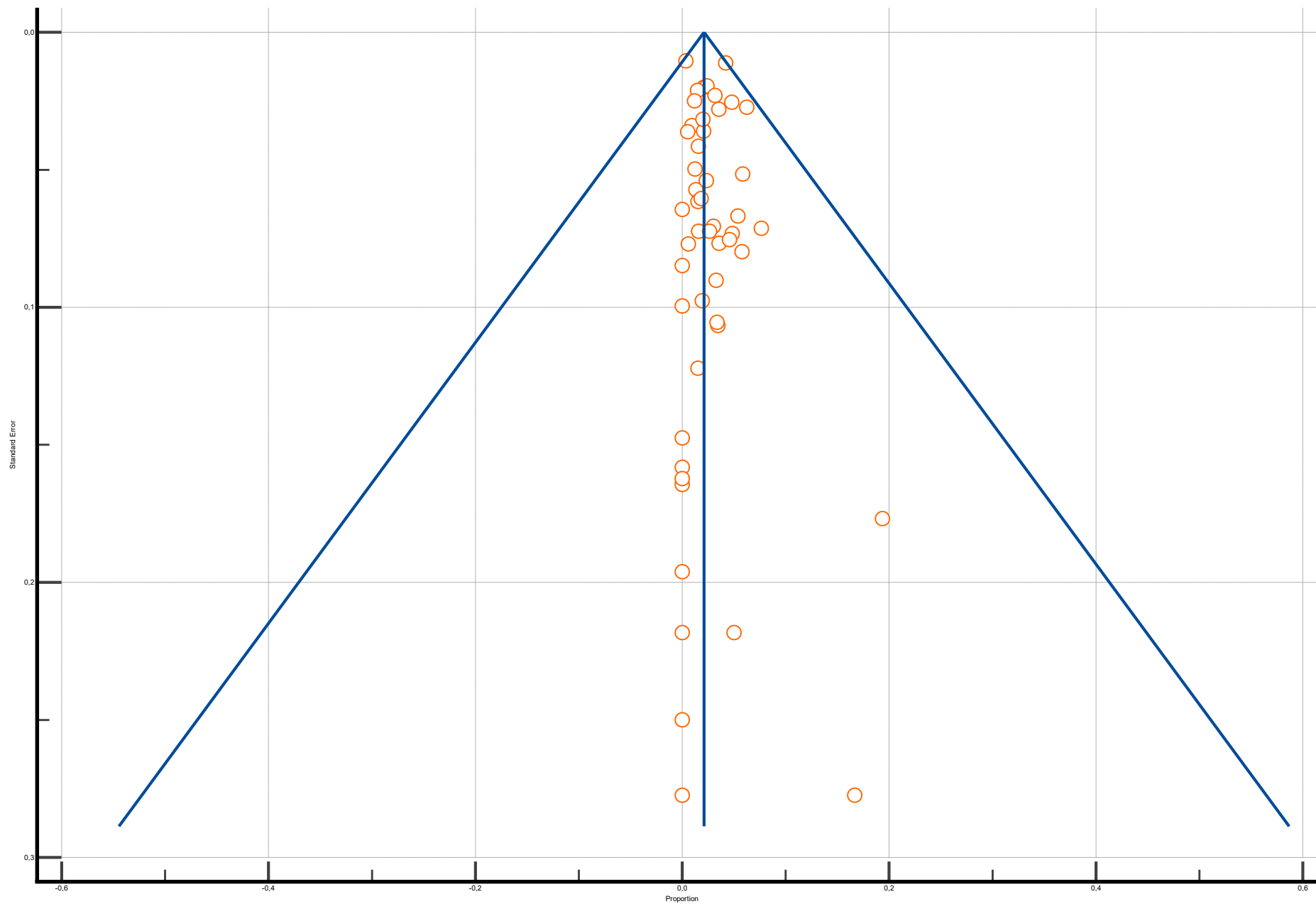

Supplement: Supplementary file 1 [file jpm-13-01429-s001.zip › Supplementary material S17.pdf]

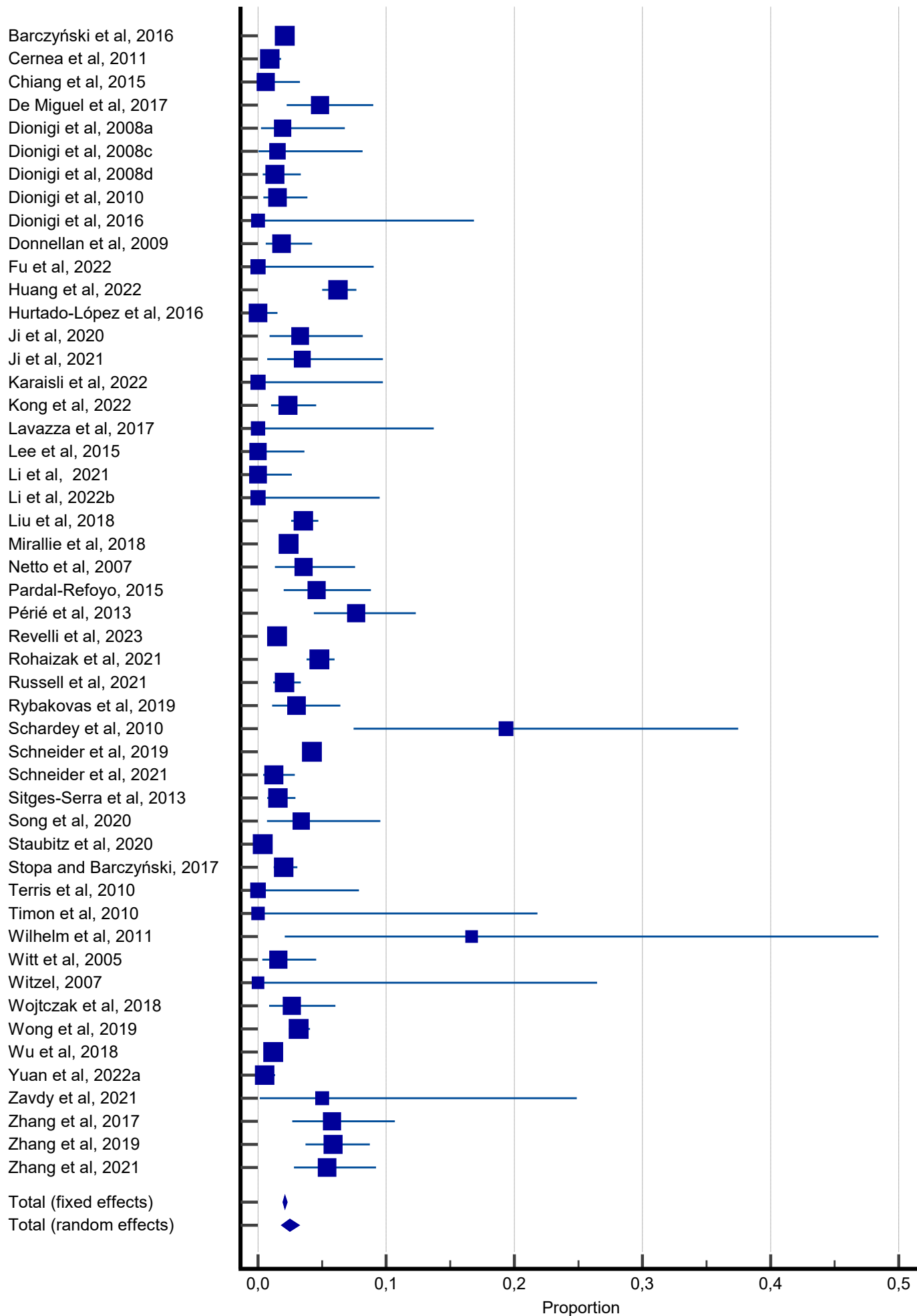

Supplement: Supplementary file 1 [file jpm-13-01429-s001.zip › Supplementary material S18.pdf]

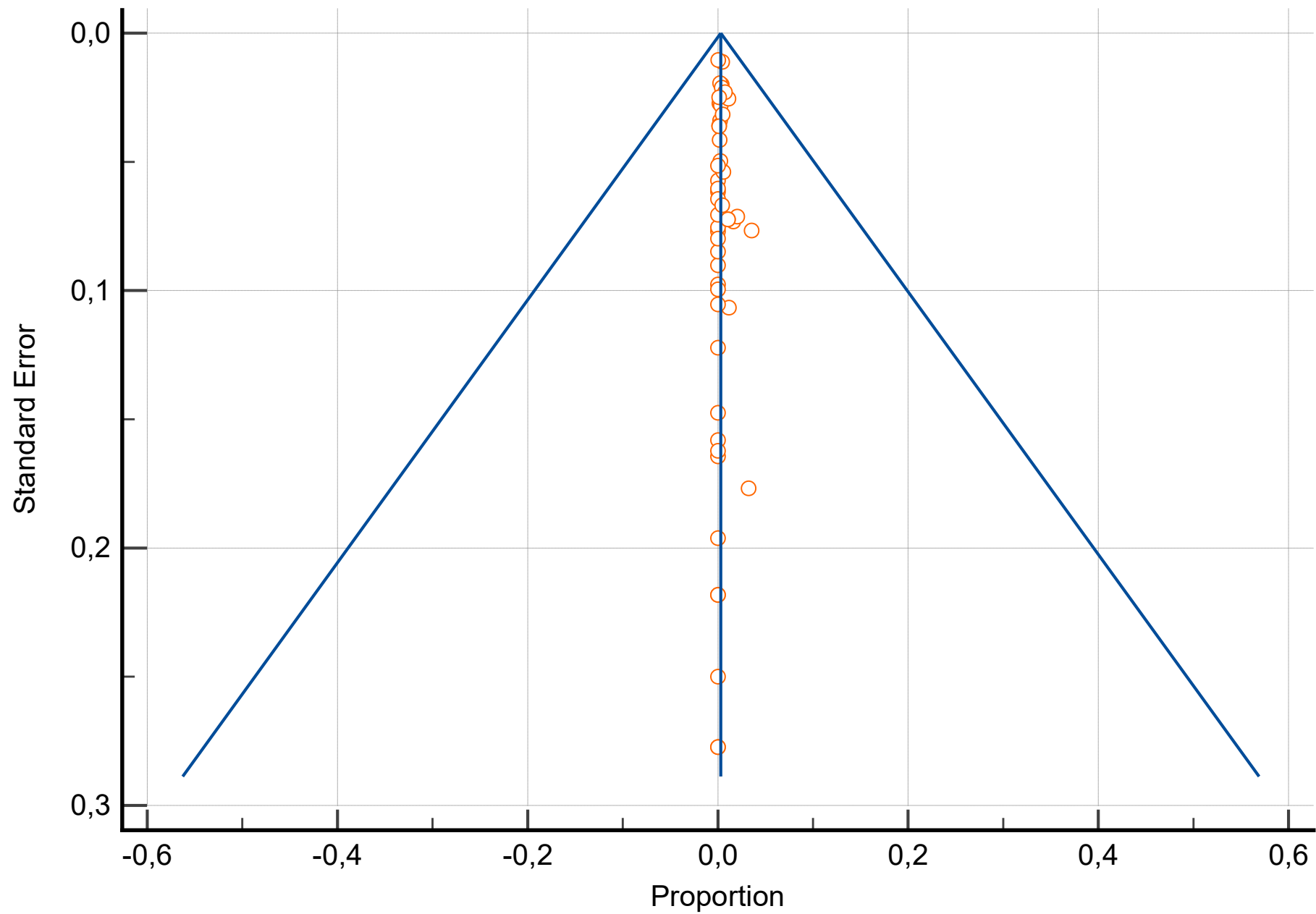

Supplement: Supplementary file 1 [file jpm-13-01429-s001.zip › Supplementary material S19.pdf]

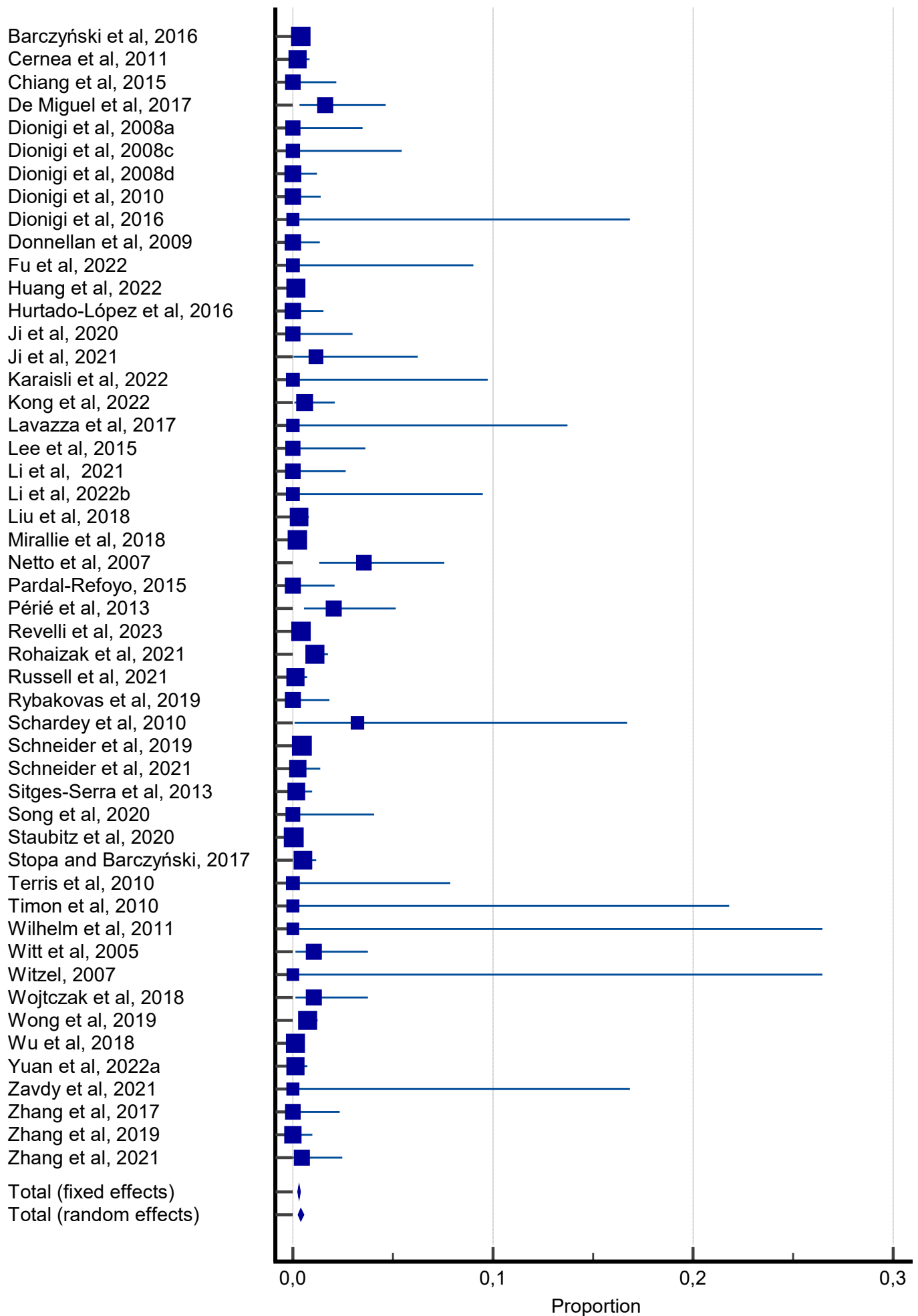

Supplement: Supplementary file 1 [file jpm-13-01429-s001.zip › Supplementary material S20.pdf]

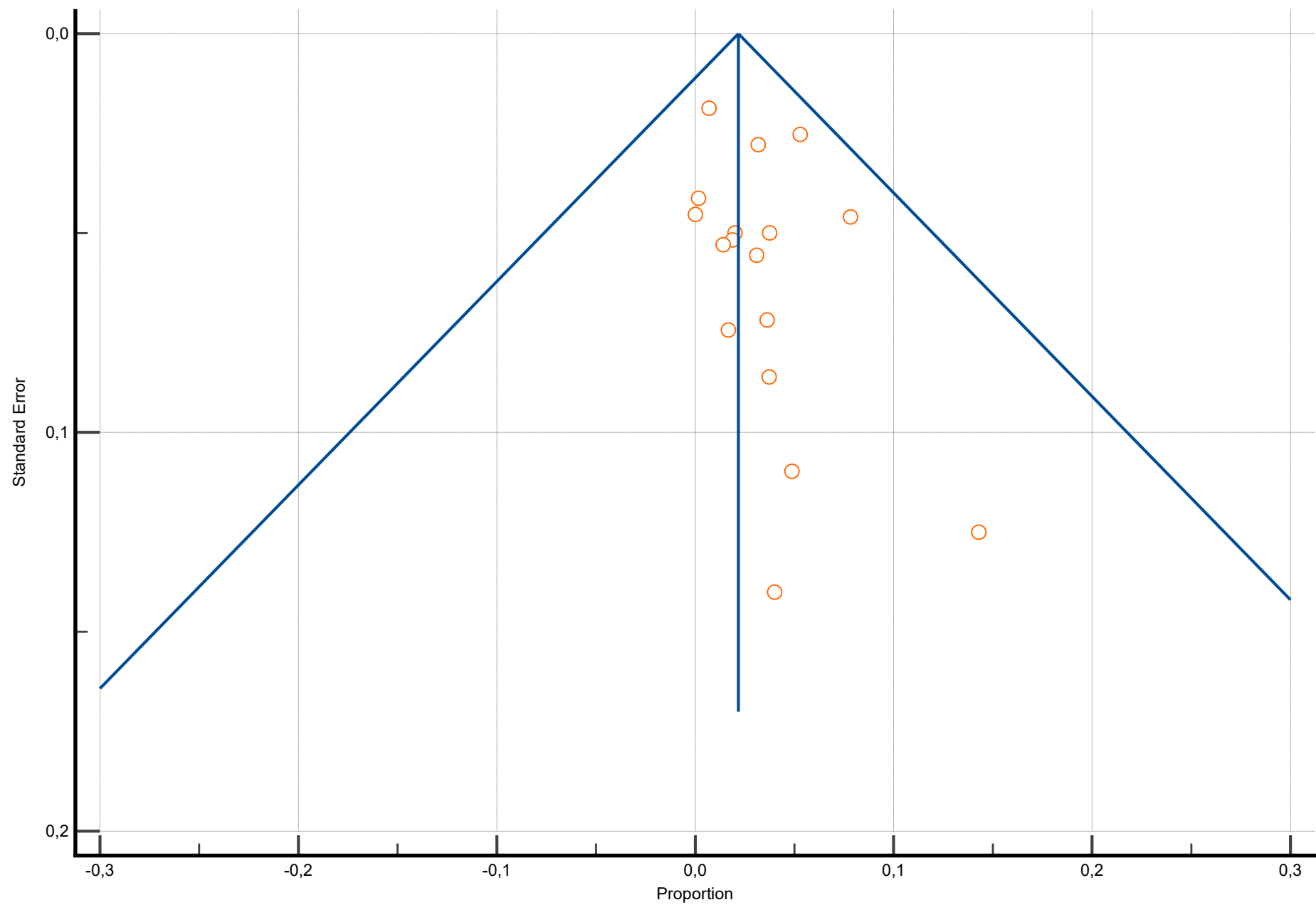

Supplement: Supplementary file 1 [file jpm-13-01429-s001.zip › Supplementary material S21.pdf]

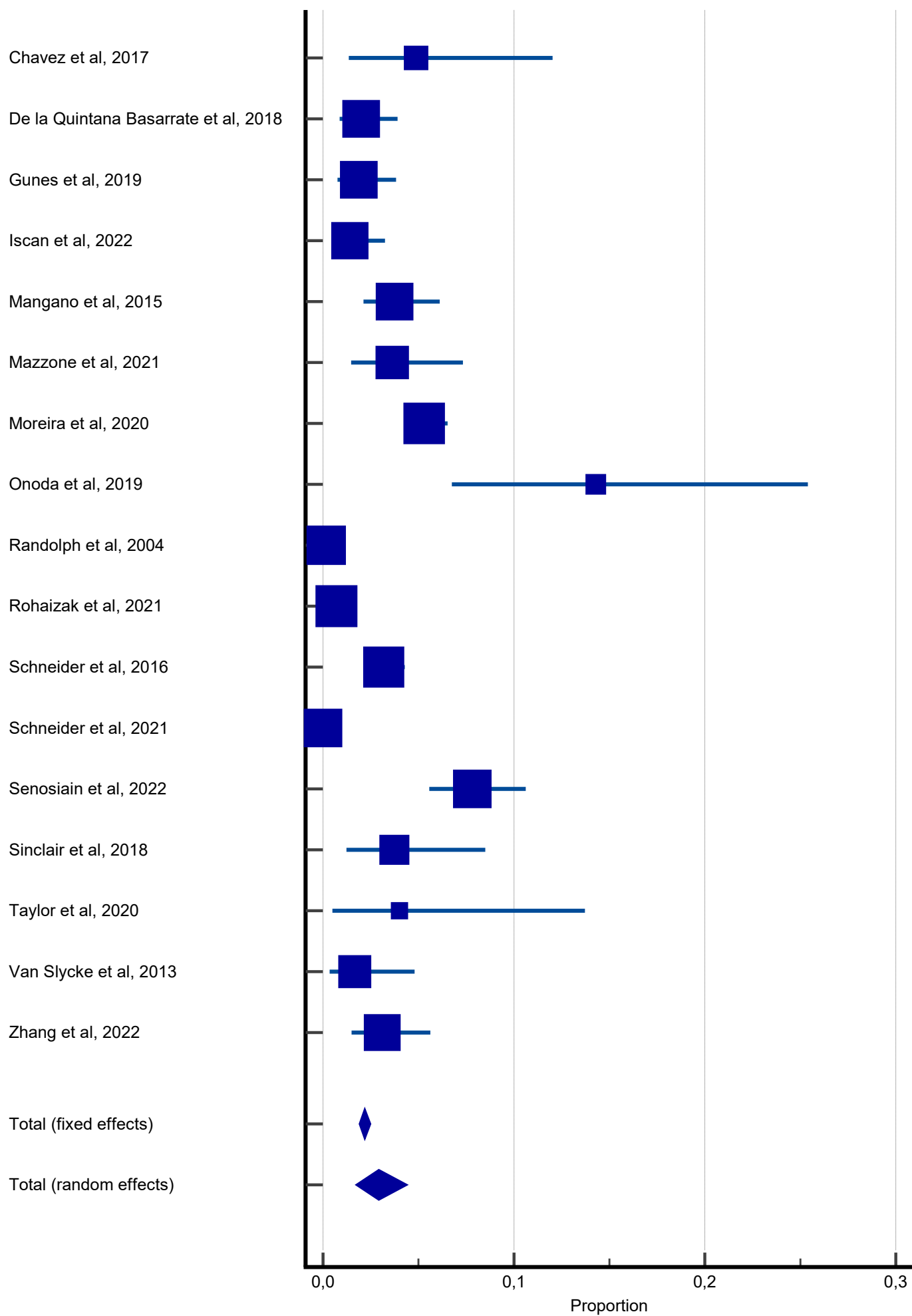

Supplement: Supplementary file 1 [file jpm-13-01429-s001.zip › Supplementary material S22.pdf]

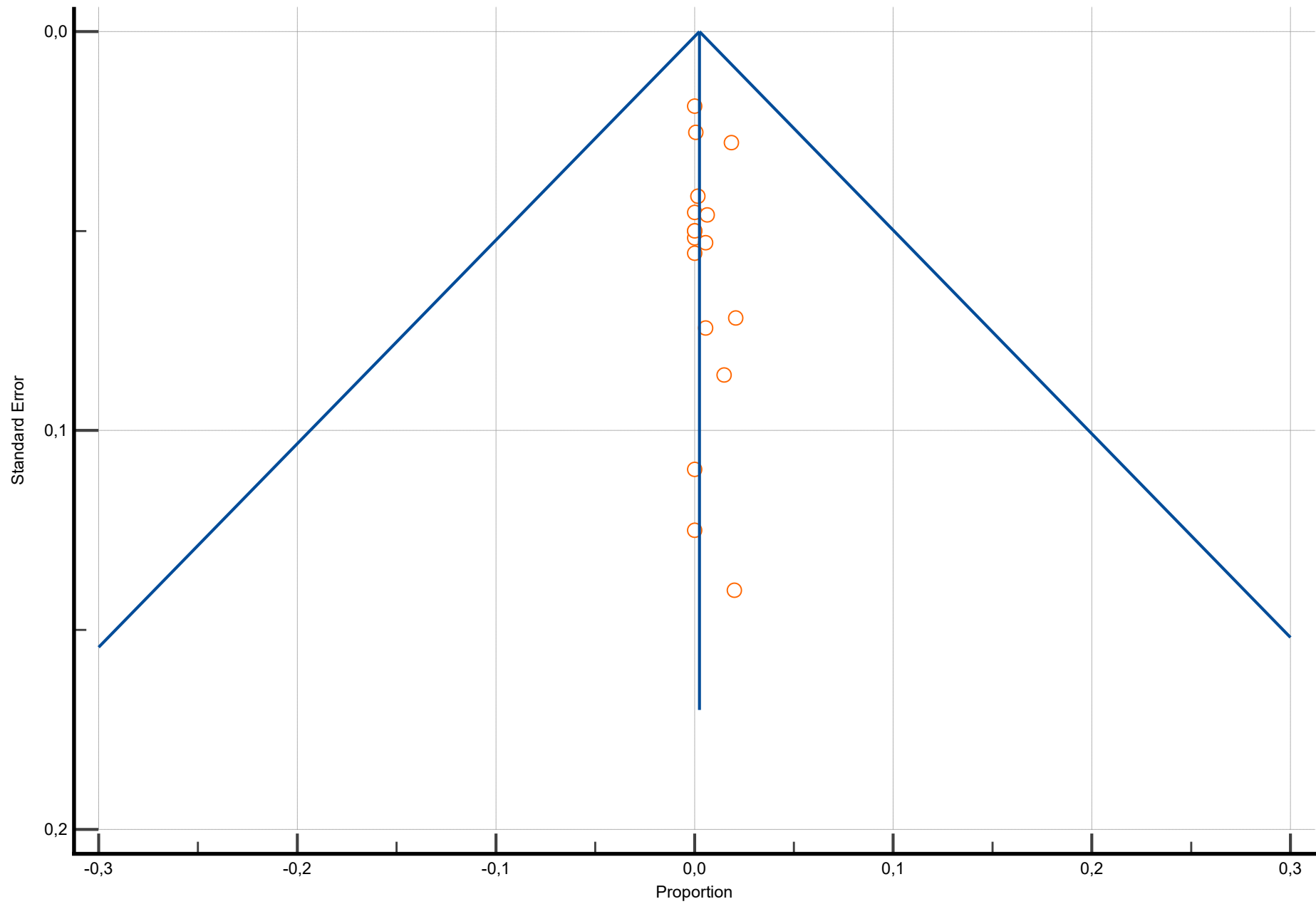

Supplement: Supplementary file 1 [file jpm-13-01429-s001.zip › Supplementary material S23.pdf]

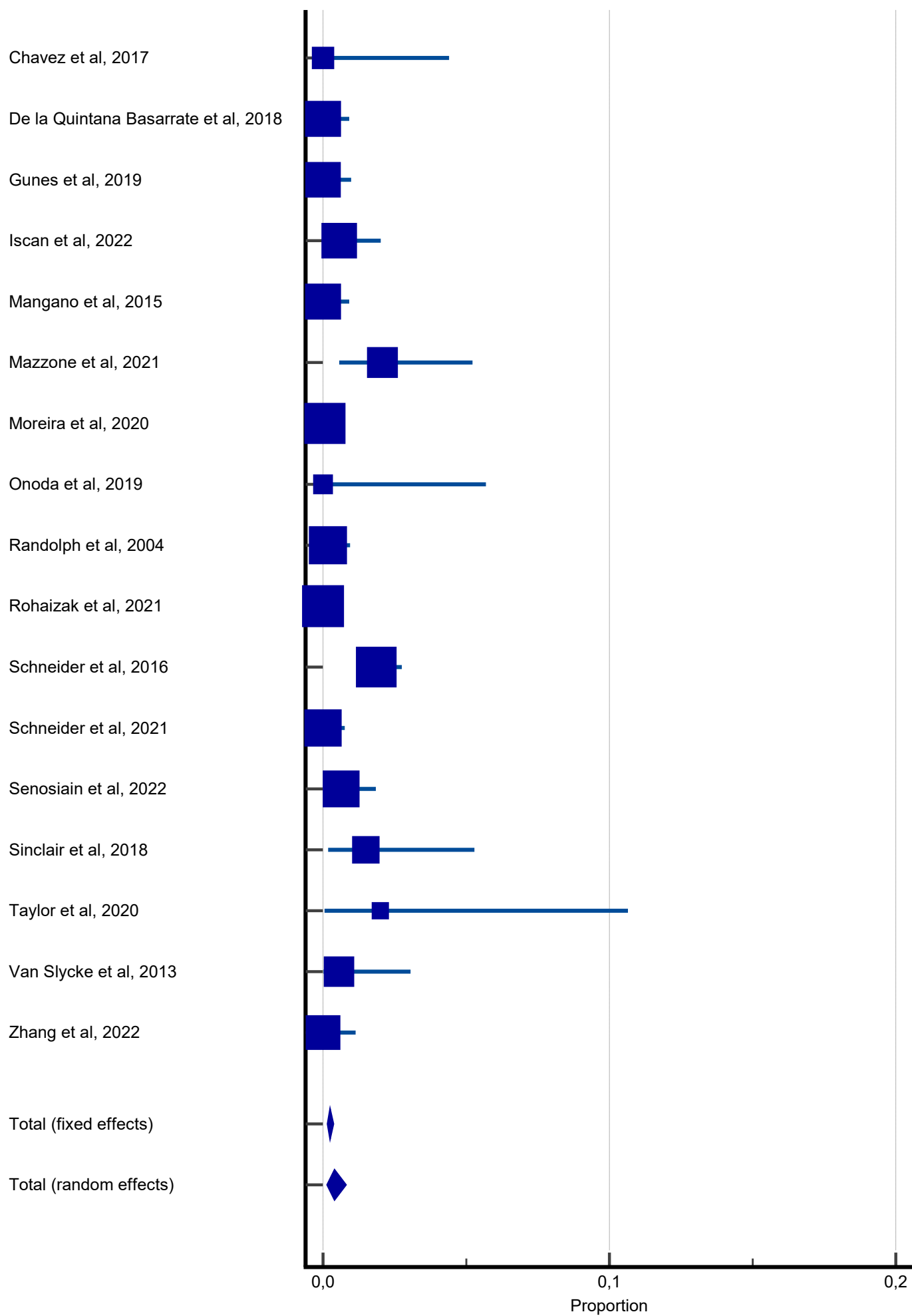

Supplement: Supplementary file 1 [file jpm-13-01429-s001.zip › Supplementary material S24.pdf]

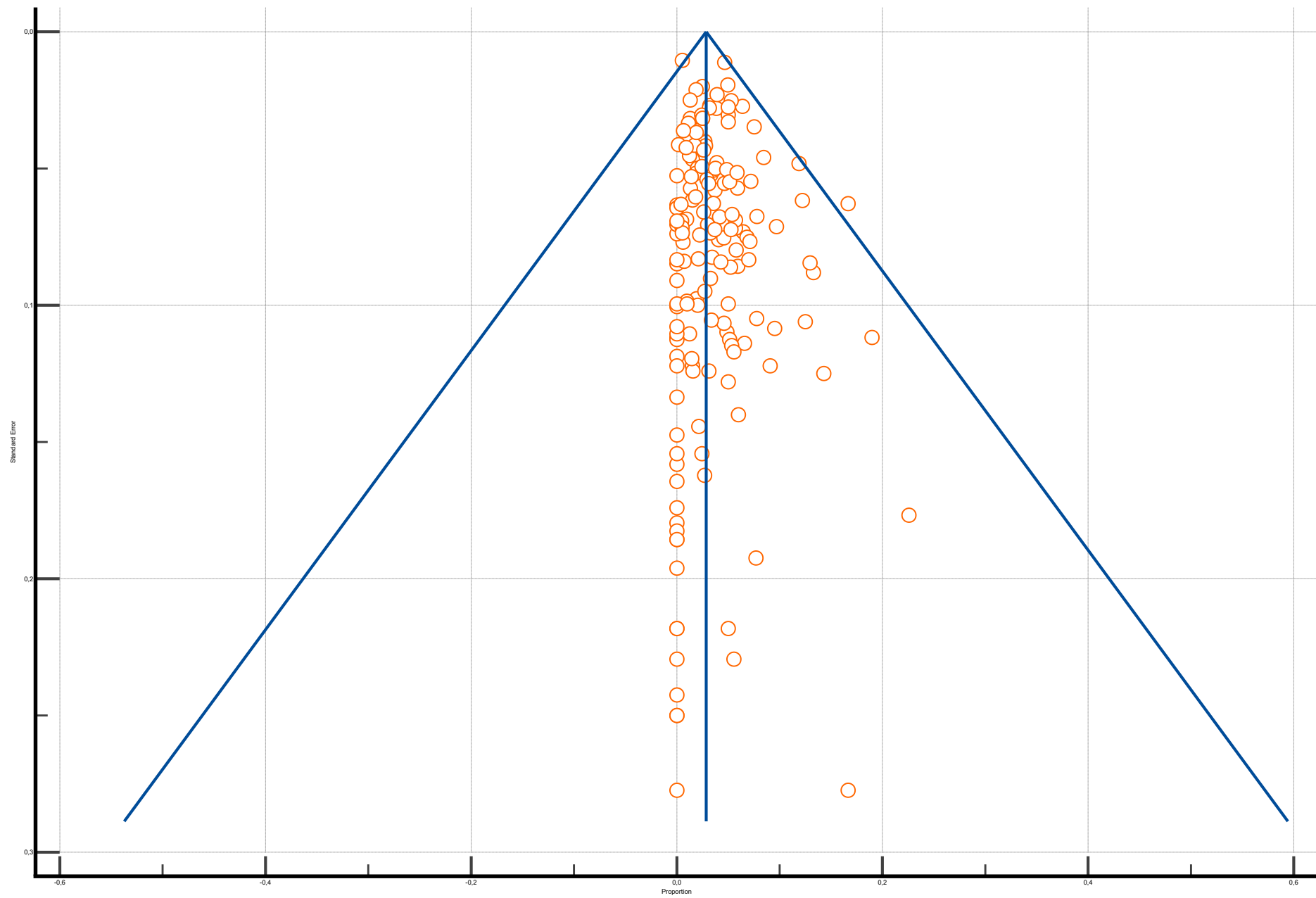

Supplement: Supplementary file 1 [file jpm-13-01429-s001.zip › Supplementary material S5.pdf]

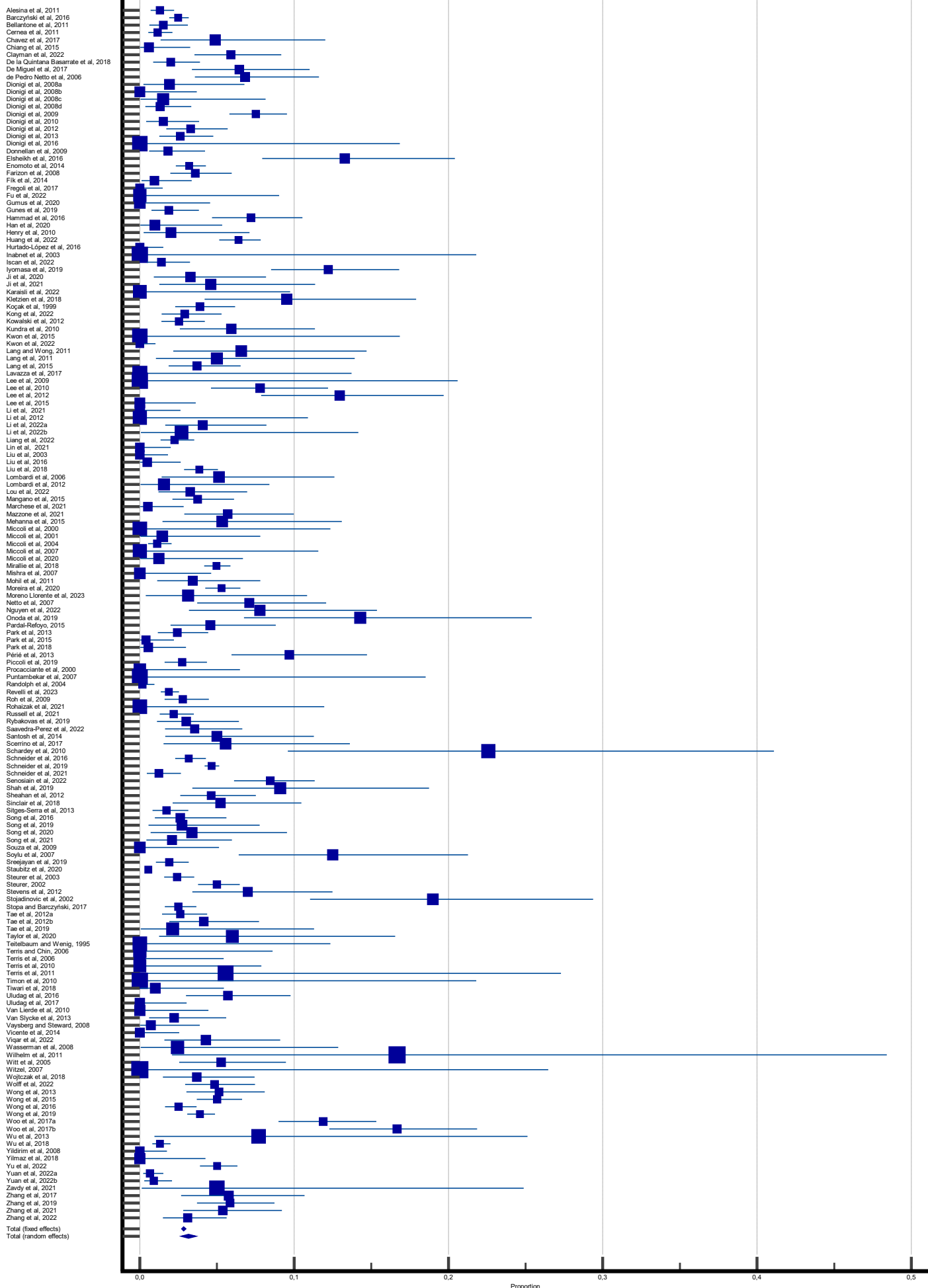

Supplement: Supplementary file 1 [file jpm-13-01429-s001.zip › Supplementary material S6.pdf]

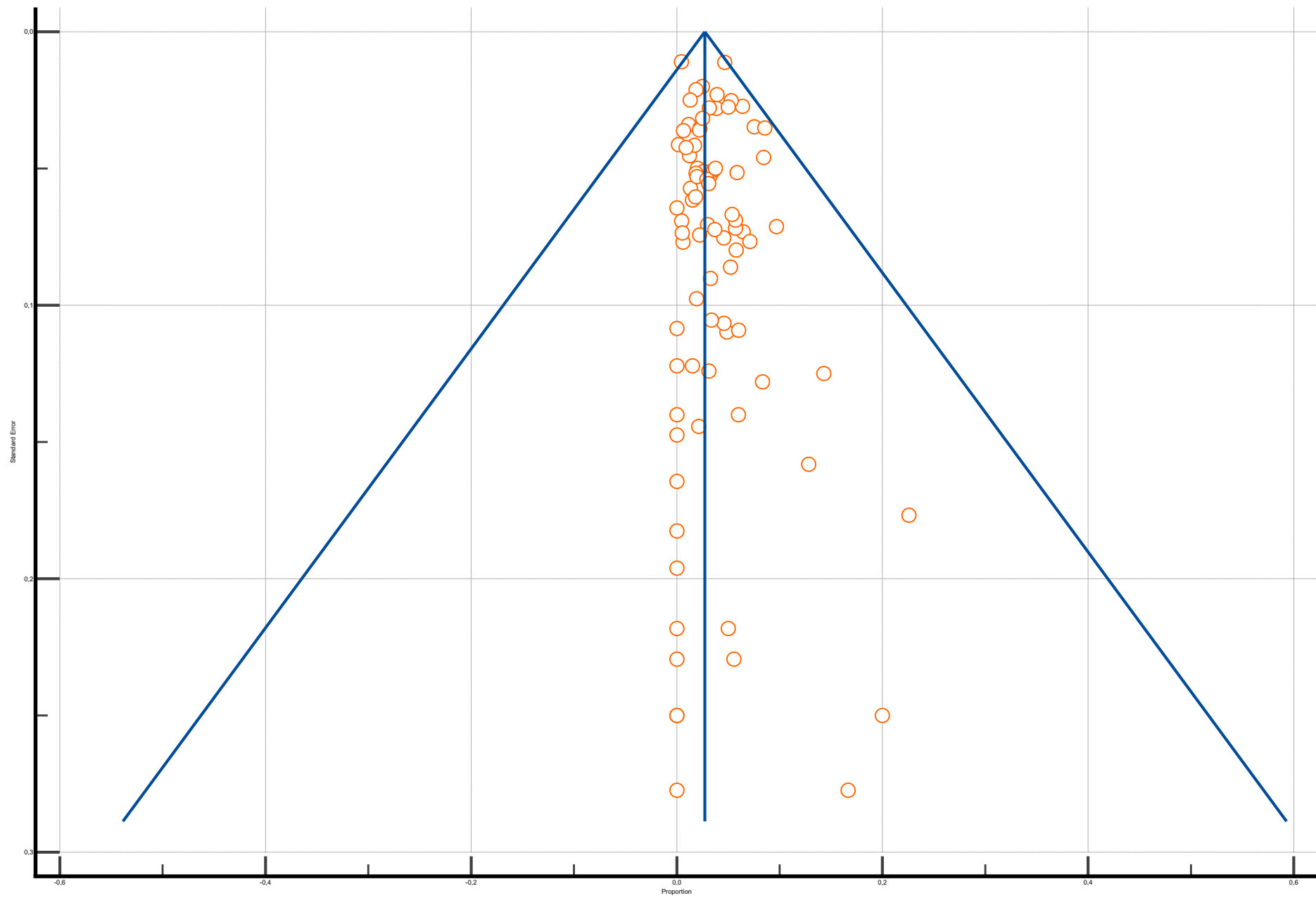

Supplement: Supplementary file 1 [file jpm-13-01429-s001.zip › Supplementary material S7.pdf]

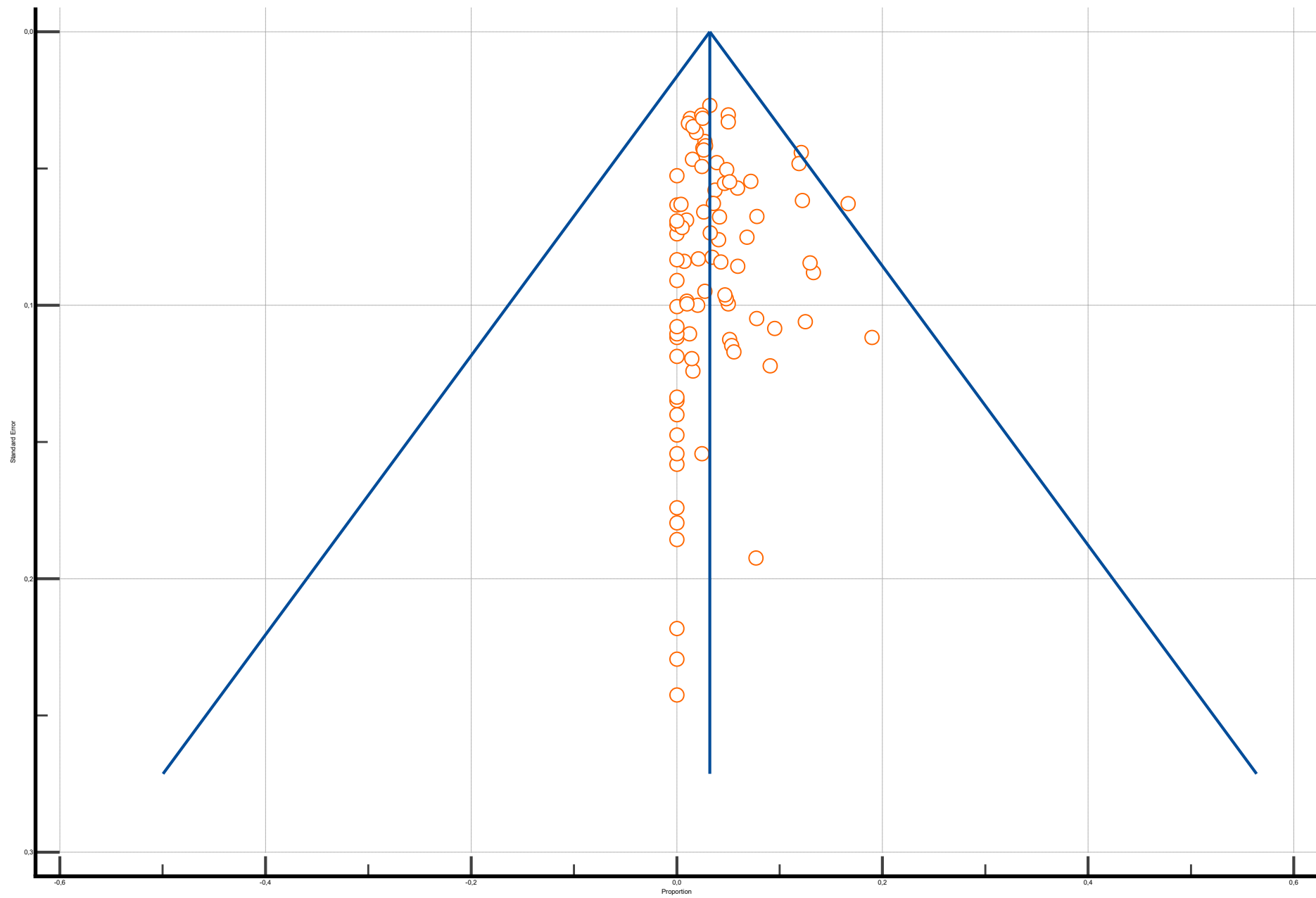

Supplement: Supplementary file 1 [file jpm-13-01429-s001.zip › Supplementary material S9.pdf]
